# An integrated method for understanding the function of macro-lithic tools. Use wear, 3D and spatial analyses of an Early Upper Palaeolithic assemblage from North Eastern Italy

**DOI:** 10.1371/journal.pone.0207773

**Published:** 2018-12-12

**Authors:** Isabella Caricola, Andrea Zupancich, Daniele Moscone, Giuseppina Mutri, Armando Falcucci, Rossella Duches, Marco Peresani, Emanuela Cristiani

**Affiliations:** 1 Department of Oral and Maxillofacial Sciences, Diet and Ancient Technology Laboratory (DANTE), Sapienza University, Rome, Lazio, Italy; 2 Department of Classics, Sapienza University, Rome, Lazio, Italy; 3 Department of Early Prehistory and Quaternary Ecology, Tübingen University, Tübingen, Baden-Württemberg, Germany; 4 MUSE, Science Museum, Trento, Trentino-Alto Adige, Italy; 5 Department of Humanities, Ferrara University, Ferrara, Emilia-Romagna, Italy; Max Planck Institute for the Science of Human History, GERMANY

## Abstract

The article presents an original analysis which combines use-wear, 3D modelling and spatial analyses to experimental archaeology in order to investigate Early Upper Palaeolithic flint-knapping gestures and techniques involving the use of macro-lithic tools. In particular, the methodological framework proposed in this paper was applied to the study of Protoaurignacian and Aurignacian macro-tools from Fumane Cave (Verona, Italy). Combining spatial analysis and use wear investigation, both at low and high magnifications, permitted the identification and detailed description of the use-related traces affecting both the hammerstones and retouchers which, at Fumane Cave, were used at different stages during flint tool production. Several experimental activities were performed including core reduction, maintenance, and blank production together with different types of edge retouching. From a methodological perspective, the protocol of analysis permitted to codify specific traces and to produce quantitative data related to their geometry and distribution over the tool’s surface, according to the activities and gestures performed. The results obtained allowed a careful investigation of the function and the gestures associated to the use of the macro-lithic tools coming from the Protoaurignacian and Aurignacian levels of Fumane Cave while providing a methodological tool for interpreting different archaeological samples.

## Introduction

Interest in the study of macro-lithic tools has increased in recent years, in relation to their potential for reconstructing the variability of adaptive human choices. First coined by Adams and colleagues [1], the term “macro-lithic tools” refers to a rather varied category of stone artefacts used for percussion, abrasion, polishing, cutting and grinding activities. The variability in the use of macro-tools in the past led to in-depth study of this category of artefacts, which has been analyzed from both a technological [2–6] and functional point of view, through the observation of macro and micro-traces [7–23]. There have also been important studies of the mechanical [24–26] and physical properties of the rocks [27], applying UBM laser profilometry methods [28], and of the residues [29–38]. Furthermore, the principles of tribology have made a great contribution to the study of macro-lithic tools for understanding the various processes that lead to use wear development [39–48].

So far, most of the functional data regarding macro-lithics comes from later prehistoric contexts–e.g. the Neolithic and Chalcolithic–while little information is available on the early use of such tools during the Palaeolithic and Mesolithic. Recent studies carried out on the tools found in the Bilancino site [49–52] and Grotta Paglicci [53], both in Italy, have emphasized the relationship between these tools and technological aspects such as plant food processing during the Upper Palaeolithic. Skills involved in the processing of different raw materials, such as plants [54–59] and minerals [60–64] have little visibility in the archaeological record. It is clear that it is necessary to intensify the functional studies on this category of artefacts, especially with regards to hunter-gatherer societies. The techno-cultural choices of these groups, for example in relation to a general evolution of human cognition and social interaction, could have been much more complex [65]. These choices encouraged the creation or the adoption of innovative technologies combined with a series of collateral activities, such as the ability to collect raw materials, transport strategies, the complementary use of tools to produce other tools, or to process organic and inorganic raw materials [66].

Tools used in percussion activities, such as spheroids and anvils, are evident since the earlier phases of the Palaeolithic [67–70], being made out of different raw materials and used to process different substances.

Macro-lithic tools are also related to the production of knapped stone tools. Indeed, hammerstones and retouchers made of stone [4,21,71–73] and bone [74,75], are found in numerous contexts, especially related to the later phases of the Palaeolithic [4]. As an example, bone retouchers have been found in different Middle and Late Pleistocene sites [76,77,78–85, 86]. Rarer are the antler billets [87–89] or wood retouchers [90].

To date, functional studies on this tool category are still lacking. Indeed, the use and the type of hammerstone or retoucher (e.g. hard or soft) is determined, or hypothesized, indirectly through the scrutiny of some morpho-metric features observed on the produced blanks (e.g. features of the impact point and the bulb, the internal and external platform angle, the dimensions of the striking platform and the morphology of the detachment scars or ridges of the dorsal face) or the retouched edge (e.g. features of scars and the bulb, the inclination of the retouch scars with respect to the opposed face, and the morphology of the scars). The identification of knapping techniques has usually been carried out in combination with experimental activities and numerous contributions have been published over the years [91–98], while more generic information is available for the use of hammers on bones [99].

Even though this type of analysis provides interesting information, some limitations do exist. Firstly, the analysis of these features focuses mainly on the knapping techniques. Secondly, it is an indirect analysis, which is exclusively based on diagnostic features on the “product”, even in those cases where the presence of hammerstones and retouchers or of ones potentially in the archaeological record would allow a detailed study of the percussion and retouching techniques. In this respect we often read about the presence of “fluvial pebbles”, which were probably used at the site as hammers, but have not been analysed by means of use wear analysis [100].

In this paper we present a multidisciplinary analysis of the repertoire of pebbles associated with the Protoaurignacian and Aurignacian levels of Fumane Cave. Such tools represent a valuable opportunity to detail the gestures of Early Upper Palaeolithic percussion activities, and the criteria involved in raw material selection and macro-lithic tool exploitation at the site. The combination of experimental archaeology, use wear analysis and GIS analysis allows further enhancement of the results provided by functional analysis, through the addition of quantitative data, and its potential has been already proved by the pioneering studies performed by De la Torre and colleagues [101], Caruana and colleagues [102] and more recently by Benito-Calvo and colleagues [103–105].

Our results further confirm the reliability of this combined methodology and provide new and relevant insights regarding the variety of percussion activities performed during the early Upper Palaeolithic occupation of Fumane Cave.

### The archaeological context: The Protoaurignacian and Aurignacian at Fumane Cave

**Fig 1:**
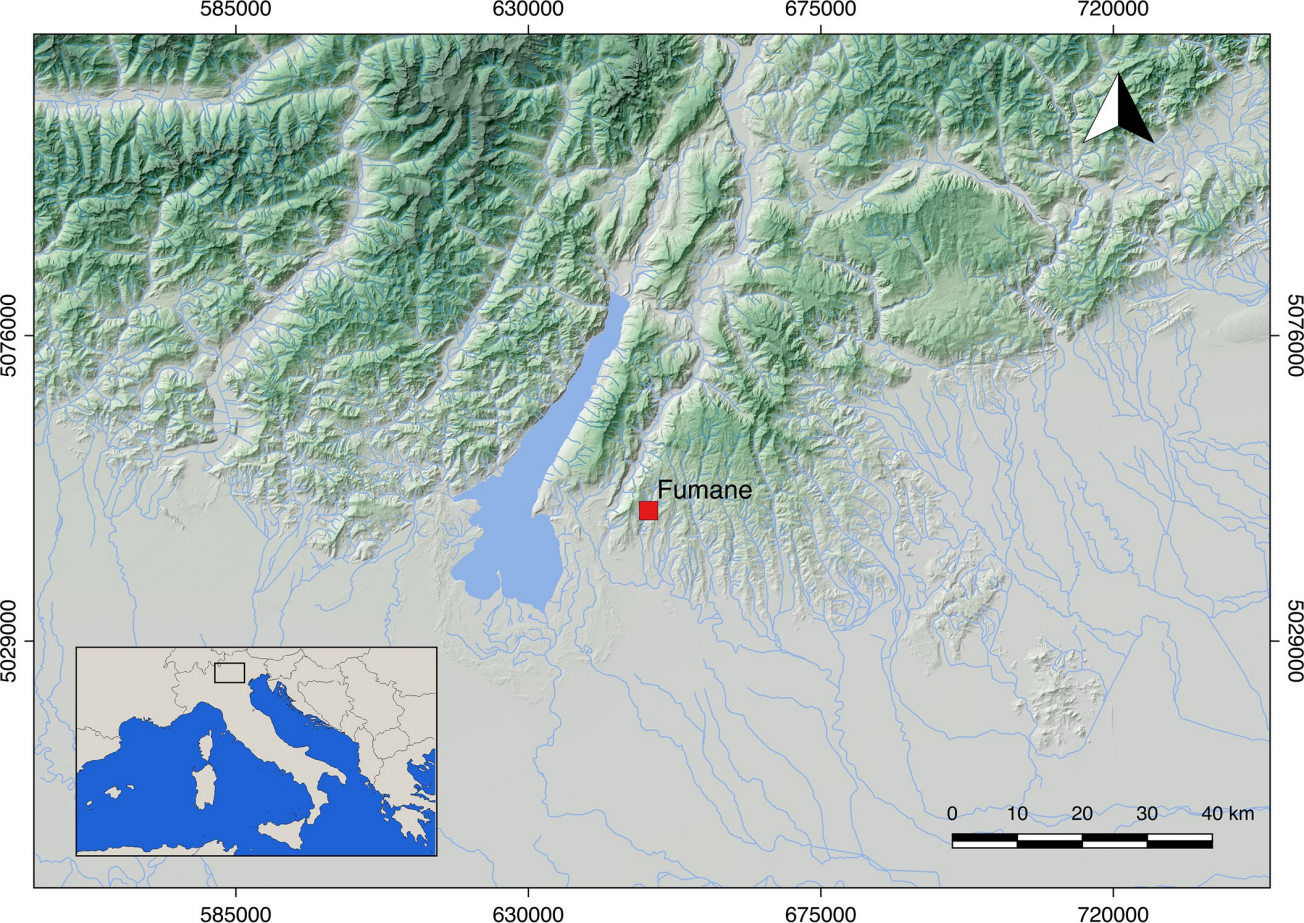
Map showing the localization of Fumane Cave (Verona, Italy).

Fumane Cave is located in the Venetian Prealps (north-eastern Italy) (**Fig 1**). The cave has been under excavation since 1988 and is characterized by a high-resolution stratigraphic sequence [106,107] spanning the Mousterian [108], Uluzzian [109], Protoaurignacian [110,111], and Aurignacian [112]. Today, it represents a key site for understanding the complex processes that led to the demise of Neanderthal populations and the spread of modern humans across Europe [113]. Layers A2 and A1 date the appearance of the Protoaurignacian to 41.2–40.4 ky cal BP, while a combustion feature embedded in the stratigraphic complex D3 dates the youngest Aurignacian phase to 38.9–37.7 ky cal BP [114]. A recent assessment of the Protoaurignacian [111,115] and Aurignacian [116] lithic technologies, has permitted an accurate narrative of the diachronic changes that occur throughout the stratigraphic sequence and enables us to critically address the techno-typological signature of the Aurignacian in northern Italy. Overall, bladelets were the first goal of the lithic production in all the studied assemblages. They were obtained from a broad range of independent reduction strategies, among which carinated technology seems to increase towards the top of the sequence. The rather standardized reduction procedures, reconstructed from the study of blanks and initial and exhausted cores, were tailored for the production of regular and frequently pointed bladelets by means of unidirectional convergent knapping progressions. Blades represented the second goal of the lithic production system and their frequency remains stable throughout the sequence. Blades were obtained from sub-prismatic cores using direct marginal percussion on flat striking platforms and were also produced during several maintenance operations carried out on bladelet cores. Unlike blades, flake production increases in the Aurignacian assemblages, where it also appears to be more standardized [117]. Tool assemblages are dominated by retouched bladelets, with frequencies that progressively decrease from layer A2 (around 80%) to the top of layer D3 (around 50%). Modification is in most cases marginal, semi-steep, and was conducted to shape bladelets with convergent retouch and bladelets with lateral retouch [118]. In both cases retouch delineation is regular and generally follows the initial morphology of the blank. Among common tools, laterally retouched blades and burins are more prevalent in the Protoaurignacian layers, while endscrapers significantly increase in the Aurignacian assemblages. Laterally retouched blades present unilateral or bilateral retouches. Modification is in most cases direct and, especially on the thicker blanks, has a scaled morphology. The so-called Aurignacian retouch [119] is instead rare. Endscrapers, both on blade and flake, display in most cases a thin working edge shaped by short lamellar removals. Some of them were made on retouched blanks. The working edge was frequently reshaped, and several wear traces were identified. Finally, thick endscrapers, such as carinated and nosed forms, were in most cases used as cores for the extraction of small and curved bladelets.

## Materials and methods

### The archaeological sample

**Fig 2:**
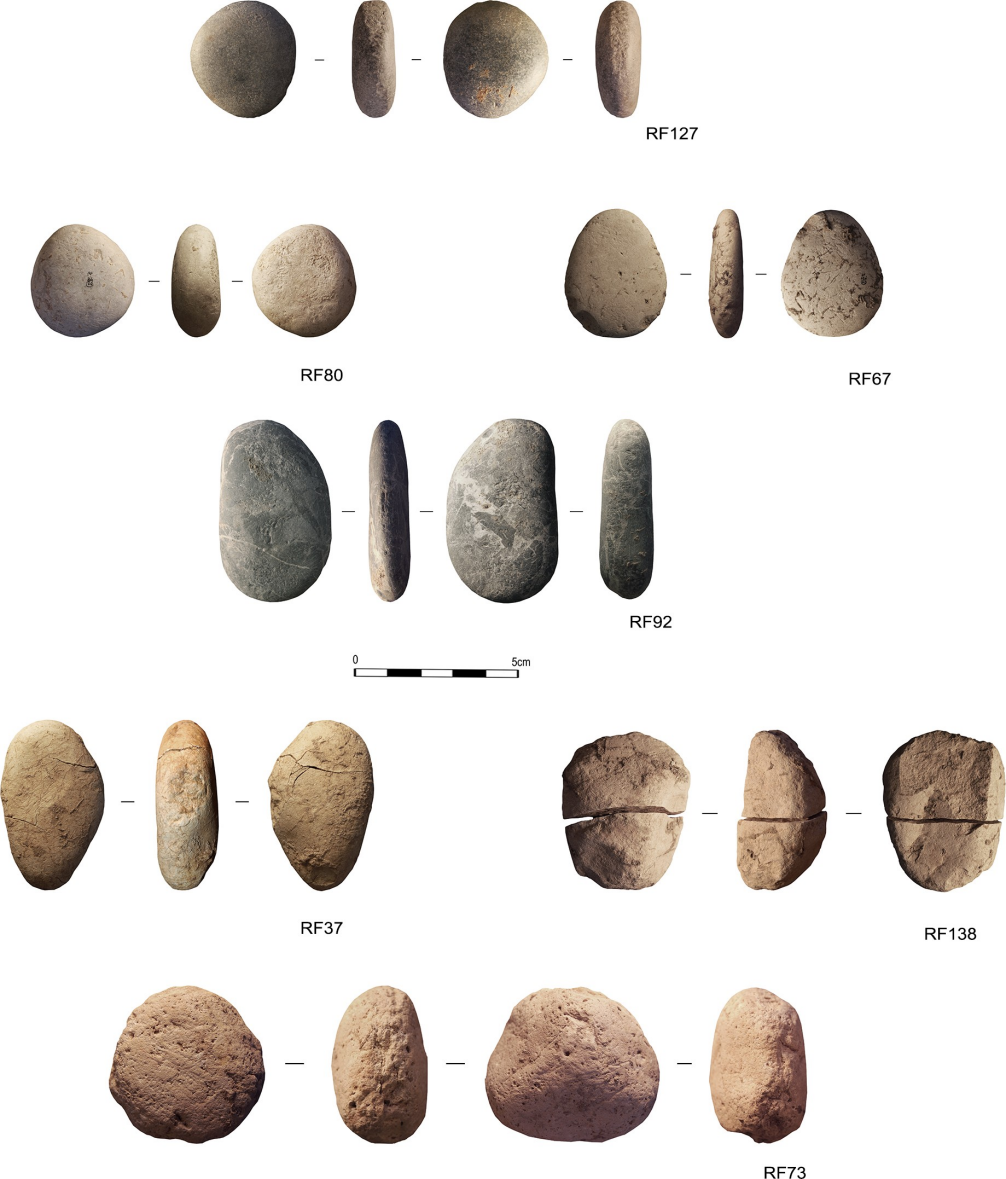
The Protoaurignacian and Aurignacian pebbles discovered in Fumane Cave (Verona, Italy).

The archaeological sample coming from the Protoaurignacian and the Aurignacian levels of Fumane Cave, is composed of 7 specimens, that characterize the entire assemblage (General Inventory Number VR67993) (**Fig 2**). These are naturally rounded pebbles originated in a fluvial sedimentary context. As suggested by previous studies [120] pebbles with a high degree of rounding have been collected, more likely, from fluvial deposits originating from high-energy water courses, like the Adige river which currently flows 20km south of Fumane. Indeed, they do not present any technological modification, their morphologies are rather recurrent, circular or oval with oval section. The overall dimensions are small, the average length equals to 68 mm, with an average width of 56 mm and an average weight of 322 gr.

Pebbles are made of sedimentary and metamorphic rocks: a) compact limestone, with fine texture (n 4); b) soft limestone, with a characteristic pink and white colour (n 2); c) ophicalcite, a metamorphic rock with carbonate cement veins of allochthonous origin (n 1) (Determination by Stefano Bertola).

However, as stressed by Bertola and colleagues [120] in the case of sedimentary rocks, the lithologies are various, attributable to different horizons included in the carbonatic formations cropping in the area, from Upper Cretacic Scaglia Rossa to Jurassic “Calcari Grigi”

Within the archaeological sample, 6 artefacts are intact or with perfectly reassembling parts, while 1 sample are fragmentary, along with one specimen characterised by fractures caused by a probable source of heat that caused it to expand (**Table 1**). No permits were required for the artefacts’ study as one of the authors (MP) is Director of the excavation at the site of Fumane Cave and responsible for the scientific activity carried out on the archaeological findings recovered from the site. Regular permits have been received (ref. DG-APAB4646) for all aspects of this work from the archaeological authority, the Soprintendenza Archeologia, Belle Arti e Paesaggio per le Province di Verona, Rovigo e Vicenza (SAPAB—VR).

**Table 1:** Information on archaeological sample. US, dimensions, raw material, integrity, colour and morphology.

US	ID	Length (mm)	Width (mm)	Thickness (mm)	Weight (g)	Integrity	Raw Material	Colour	Morphology
D3	RF73	66	71	42	364.4	Intact	Soft Limestone	Pink	Circular/Oval Section
D3	RF138	69	99	75	206.8	Fragments (n.2)	Soft Limestone	Pink	Sub-Oval/Plane-Convex Section
D3	RF37	85	48	26	188.9	Alteration	Limestone	Brown	Oval/Oval Section
D3+D6	RF92	98	55	22	246.3	Intact	Ophicalcite	Grey/White veins	Oval/Oval Section
D6	RF80	48	43	18	81.8	Intact	Compact Limestone	White	Circular/Oval Section
A1	RF67	73	51	15	119.0	Intact	Compact Limestone	Brown	Oval/Oval Section
A2	RF127	52	43	16	88.6	Intact	Compact Limestone	Brown	Circular/Oval Section

### Use wear analysis

The artefacts were analyzed applying a functional approach along with the design and application of a dedicated experimental framework. The functional approach is based on the analysis of different aspects related to the use of macro-lithic tools.

For the study, the specimens were observed utilizing a Zeiss Axio Zoom V16 binocular stereo microscope, oculars PI 10x/23, objective 1x/0.25 FWD 56mm, with progressive magnifications ranging between 10x and 80x. This low-magnification observation allowed us to propose a hypothesis regarding the gestures and details related to the kinetics of the object. Furthermore, it allowed us to determine the nature and status of the processed matter with which the object came into contact. Topography and microtopography, grain shapes, pits, striation and fracture morphologies on experimental and archaeological artefacts have been described according to parameters already described in literature [3,20]. 3D models of the surface were produced utilizing Mountain Map Premium 7.2, which provided more information related to the evolution of the microtopography and details concerning the morphology of the identified traces.

A second level of observation consisted of the analysis of the specimens at higher magnification (50-500x) using Zeiss Scope A.1 metallographic microscope equipped with 10x oculars and with objectives ranging from 5x to 50x. This allowed the investigation of micro wear (e.g. micro-striations and micro-polishes) to achieve more information about the use of the tools. Polishes have been described by taking into account their texture, topography, distribution, extension and linkage (for more details see [23,121,122]). The surfaces have been documented using a Zeiss Axiocam 305/506 color camera and were washed with neutro phosphate detergent (Derquim) and ultrasonic cleaner AU-32 (ARGO LAB) for 15/20m.

### Photogrammetry

3D Models of both experimental and archaeological samples have been created through the application of photogrammetry. Following the protocol developed by Porter and colleagues [123] 3D models of the artefact were built using Agisoft Photoscan 1.3.4.

The tools were placed on an automatic turntable in order to produce 360° sets of each of the tool’s surfaces. Pictures of the tools were shot using a Nikon D7200 DSLR camera equipped with a Nikkor 105 Macro Lense. Each picture was taken every 15°, and at every full revolution the camera was lifted and slightly titled towards the target for a total of 72 picture per object side. A total of 144 pictures were taken per object, which were subsequently imported in Agisoft Photoscan Pro 1.3.4 to produce high quality dense point clouds and meshes.

### Surface morphometric analysis

GIS analysis has been adopted to analyse the morphometric characteristics of both experimental and archaeological samples. Applying both the methodologies proposed by Caruana et al [102], Benito-Calvo et al [103] and de la Torre et al [101] it has been possible to analyse and quantify use wear patterns originated from both retouching and percussive activities. After the creation of 3D Models, Digital Elevation Models (DEM) featuring a resolution of 0.5mm were created in Agisoft Photoscan 1.3.4 and imported as raster files in ArcGIS 10.5.

Digital Surface Models were generated in order to analyse the topographic features characterising the tool’s surface. At first a Hillshade model of the entire surface was created. This allowed a first morphometric assessment of the surface topography that permitted the identification of the Functional Area/s (FA) of the surface which are affected by use.

Once identified, the FA of the tool was extracted from the original DEM as a new raster surface and three kinds of Digital Surface Models (DSMs) were generated to identify and interpret use wear.

Slope, which identifies the rate of change in the z-value from each of the cells composing a raster surface allows the identification of changes in the surface elevation such as depressions or pits characterising the objects FA. Subsequently, two DSMs devoted to the analysis of surface roughness were generated. Analysing surface roughness permits the analysis of the degree of homogeneity or heterogeneity characterising the tool’s surface. As already stated by Benito-Calvo and colleagues (2015) the measurement of surface roughness can lead to the identification of polished areas (low roughness) generated by use. Two methods of surface roughness measurement have been applied: *Terrain Ruggedness Index (TRI)* and *Vector Ruggedness Measure (VRM)*. *TRI* is based on the algorithm proposed by Riley and colleagues [124] and calculates the sum change in elevation between a grid cell and its neighbourhood. In the resulting DSM, a TRI value of 0 represents the minimum degree of roughness (i.e. homogeneous surface). *Vector Ruggedness Measure (VRM)* measures roughness as the dispersion of vectors orthogonal to the surface within a specific neighbourhood. This method captures variability in slope and aspect into a single measure. A value of 0 represents no terrain variation (or lowest roughness) while a value of 1 represents a complete terrain variation (maximum roughness). In the case of the experimental replicas, 3D models and resulting DSMs were made before and after use. This allowed the mapping and quantification of the degree of variation in surface topography related to each of the experimental activities performed.

Following the methodological framework proposed by Caruana et al [102] the FAs of both experimental and archaeological implements were analysed through Topographic Position Index in order to identify areas of high micro topographic roughness coinciding with use related damage.

**Fig 3:**
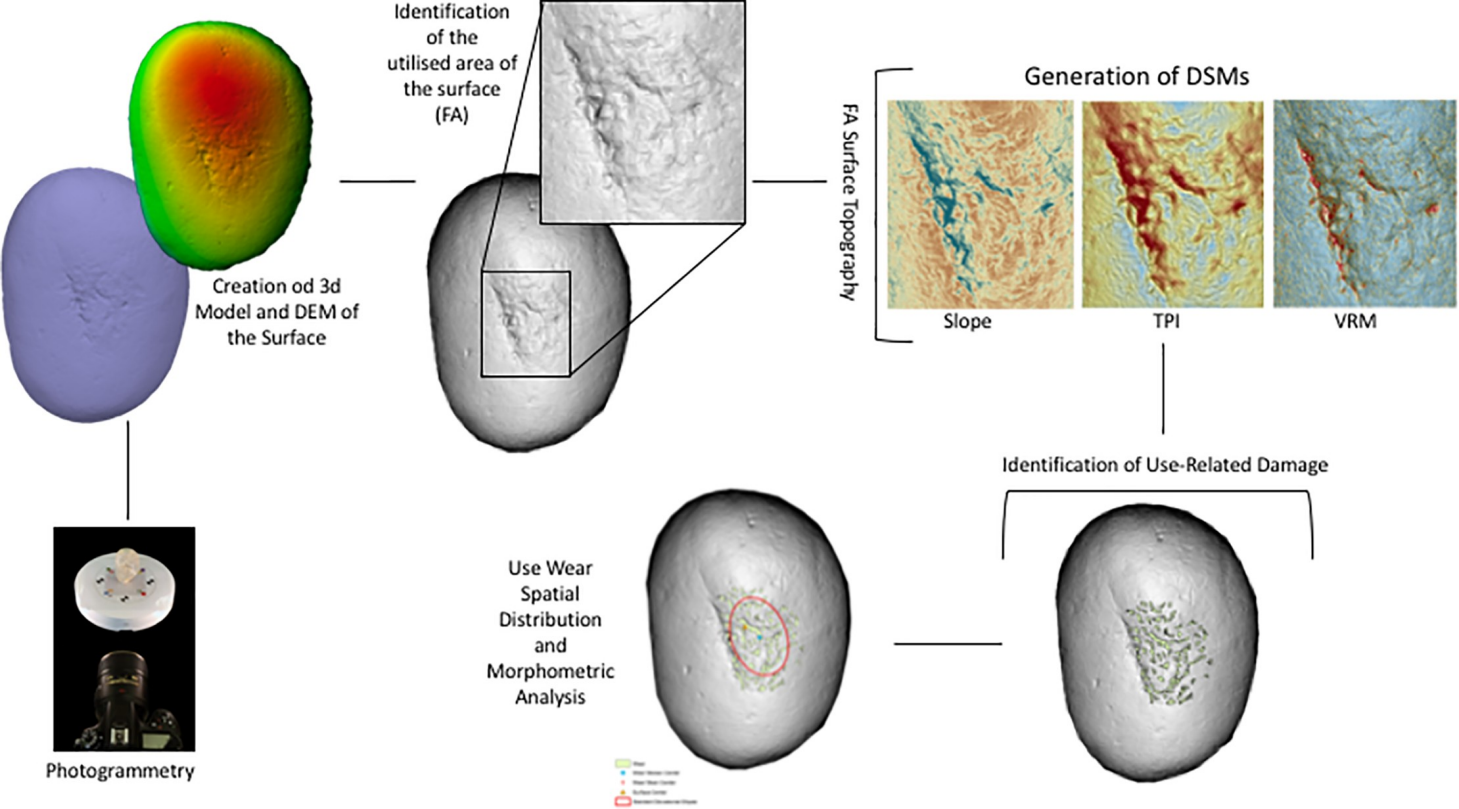
Schematic representation of the methodology applied for the creation of 3D models and the spatial analysis of the utilised areas of the tools.

*Topographic Position Index (TPI)* is an elevation residual analysis which is applied to identify depressions and ridges affecting the artefacts surface topography [103]. The DSM generated is based on the computation of the difference between the elevation of a cell and the mean elevation in a neighbourhood surrounding that cell. Neighbourhood mean elevation is calculated using a moving window centred on the cell of interest. TPI positive values indicate that the cell is higher than its neighbourhood while negative values indicate the cell is lower, corresponding to either ridges and depressions. Hot Spot Analysis (Getis-Ord GI*) was performed on the generated surface in order to identify clusters of pits and ridges highlighted by TPI and corresponding to wear caused by use. The patterns identified through Hot Spot Analysis (Getis-Ord GI*) were then transformed into polygons, which provided metric data (e.g. area, perimeter) to be statistically compared (**Fig 3**).

### Experimental framework

A dedicated experimental reference collection was necessary in order to understand the use of macro-lithics at Fumane cave, and to isolate specific gestures involved in percussion activities. The experimental framework consisted of different stages. Raw materials were collected according to the size and morpho-metric features of the archaeological specimens. Small and rounded pebbles (mean length 50 mm) of compact limestone were gathered along the Adige river bank, about 20 km away from Fumane. Coarse limestone pebbles were collected in a stream bed close to the site. The latter showed a pink/white colour, probably due to geochemical alterations related to the particular depositional environment.

The collected items (5 retouchers, 3 retouchers/hammerstones, 9 hammerstones, 2 anvil) were used in several experimental tests) and their surface was documented using both the stereo and metallographic microscopes before and after their use, in order to observe the modifications caused by use.

After a preliminary observation of the archaeological sample, it became clear that the Fumane macro-lithics had been used in various activities related to the processing of stone and materials of a non-organic nature. The experimental framework involved 19 pebbles used as hammers in various stages of bladelet production and retouchers, according to the technical solutions known from the analysis performed on the lithic artefacts from the Protoaurignacian and Aurignacian levels of the site, in which core reduction and maintenance are illustrated along with the morpho-technical features of the laminar products and the typology of the retouched tools [110,111,115,120].

For our experimental purposes, nodules of fine-grained flints were used. Several tests have been performed by the flint-knapper, following a precise strategy: a single hammer has been used to perform a specific action with the aim of isolating the functional traces, while others have been involved in different technical gestures to produce experimental replicas showing multi-functional surfaces (a complete list of uses has been illustrated in **Table 2**).

**Table 2:** List of experimental samples used in different phases of the chipped tools production.

Exp. N°	Type	Action	Knapper	L (mm)	Wi (mm)	T (mm)	We (g)	Raw material	Morphology	Working Time	Effectiveness of the experiment	Integrity
**FRS-1**	Retoucher	Scaled retouching	Expert, right-handed	65	50	15	87	Compact grey limestone	Oval/Oval section	30m	High	Intact
**F1**	Retoucher	Scaled retouching	Expert, left-handed	65	44	25	110	Compact brown limestone	Oval/Oval section	45m	High	Intact
**F18**	Retoucher/ hammerstone	Scaled retouching; Striking platform maintenance; Core shaping	Expert, right-handed	78	43	28	131	limestone with white veins	Oval/Oval section	3h	High	Small flake removal (L.10mm)
**FRP-1**	Retoucher	Marginal/abrupt parallel retouching	Expert, right-handed	65	46	15	73	Compact grey limestone	Oval/Oval section	30m	High	Intact
**F11**	Retoucher/hammerstone	Scaled and marginal retouching / striking platform maintenance, small flakes production	Expert, right-handed	55	45	14	52	Compact grey limestone	Oval/Oval section	1h 30m	High	Intact
**FBR-2**	Retoucher/ Hammerstone	Marginal/abrupt parallel retouching; Striking platform maintenance and Bladelets removal	Expert, right-handed	49	43	28	83	Compact white limestone	Circular/ Oval section	2h	High	Intact
**F20**	Hammerstone	Striking platform maintenance	Expert, right-handed	60	29	24	65	Compact brown limestone	Oval/Oval section	45m	High	Intact
**F17**	Hammerstone	Striking platform maintenance	Expert, right-handed	68	56	28	148	Compact white limestone	Oval/Oval section	1h	High	Intact
**F10**	Retoucher	Marginal/abrupt parallel retouching	Expert, left-handed	49	38	20	56	Compact grey limestone	Oval/Oval section	2h	High	Intact
**F12**	Hammerstone	Bladelets removal; Overhang abrasion; Striking platform maintenance	Expert, right-handed	72	47	15	76	Soft pink limestone	Oval/Oval section	2h	High	Small flake removal (L.24mm)
**FSPM-13**	Hammerstone	Striking platform maintenance; Overhang abrasion; Bladelets removal	Expert, right-handed	59	35	16	54	Soft limestone	Oval/Oval section	2h	High	Small flake removal (L.23mm)
**F14**	Hammerstone	Core shaping	Expert, right-handed	103	55	51	383	Compact pink limestone	Oval/Oval section	30m	Low	5 flakes removal (L.34mm)
**F15**	Hammerstone	Striking platform maintenance; Bladelets removal; Overhang abrasion; Scaled retouching; Marginal/abrupt parallel retouching	Expert, right-handed	88	61	23	171	Compact grey limestone	Oval/Oval section	45m	Medium	Small flakes removal (L.12mm)
**F16**	Hammerstone	Bladelets removal	Expert, right-handed	88	64	30	236	Compact grey limestone	Oval/Oval section	20m	Low	Broken, Longitudinal flake (L.70mm)
**FA-8**	Anvil	Anvil for flakes removal	Expert, right-handed	80	60	23	177	Compact brown limestone	Oval/Oval section	45m	High	Intact
**F19**	Hammerstone	Bladelets removal	Expert, right-handed	52	67	33	193	Soft pink limestone	Oval/Oval section	45m	High	Flakes removal (L.50mm)
**F3**	Hammerstone	Striking platform maintenance; Overhang abrasion	Expert, right-handed	63	38	20	57	Soft pink limestone	Oval/Oval section	30m	High	Intact
**FAR-8 bis**	Anvil	Anvil for bladelets retouching	Expert, right-handed	80	64	30	236	Compact grey limestone	Oval/Oval section	30m	Low	Intact
**F20**	Retoucher	Edge abrasion	Expert, right-handed	60	50	30	200	Compact grey limestone	Oval/Oval section	25m	High	Intact

**Fig 4:**
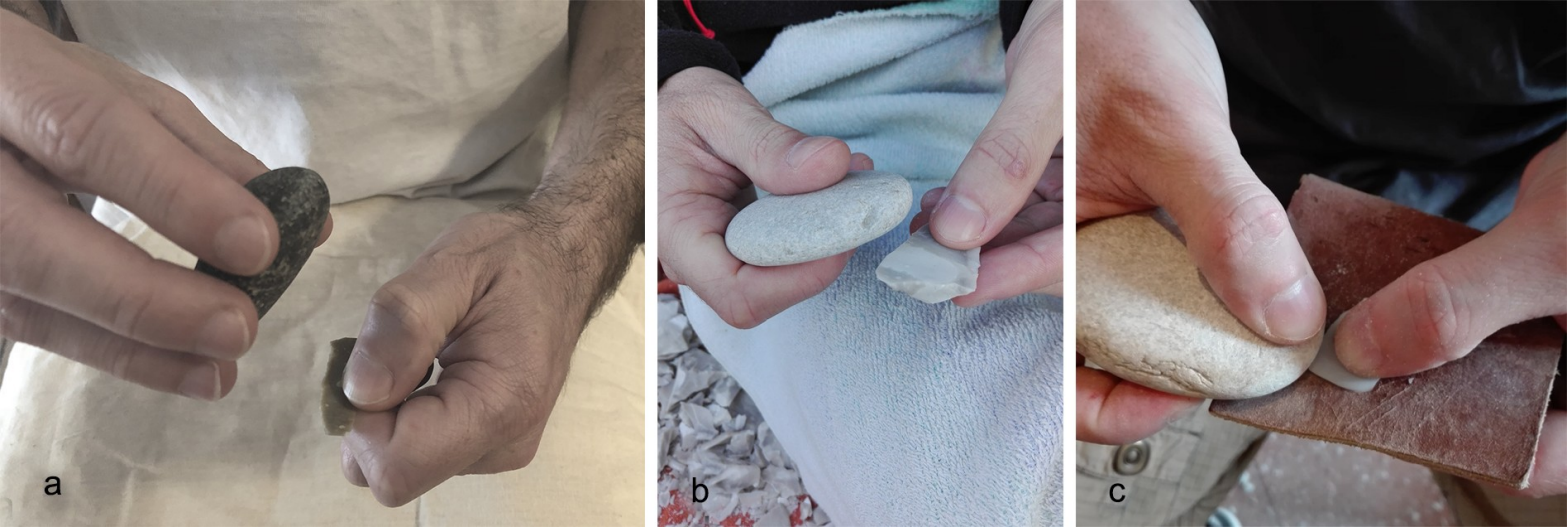
Experimental retouching. (a) Production of marginal and abrupt retouch; (b) production of scaled retouch on the lateral edge of a laminar flake; (c) blank retouch through edge abrasion.

**Fig 5:**
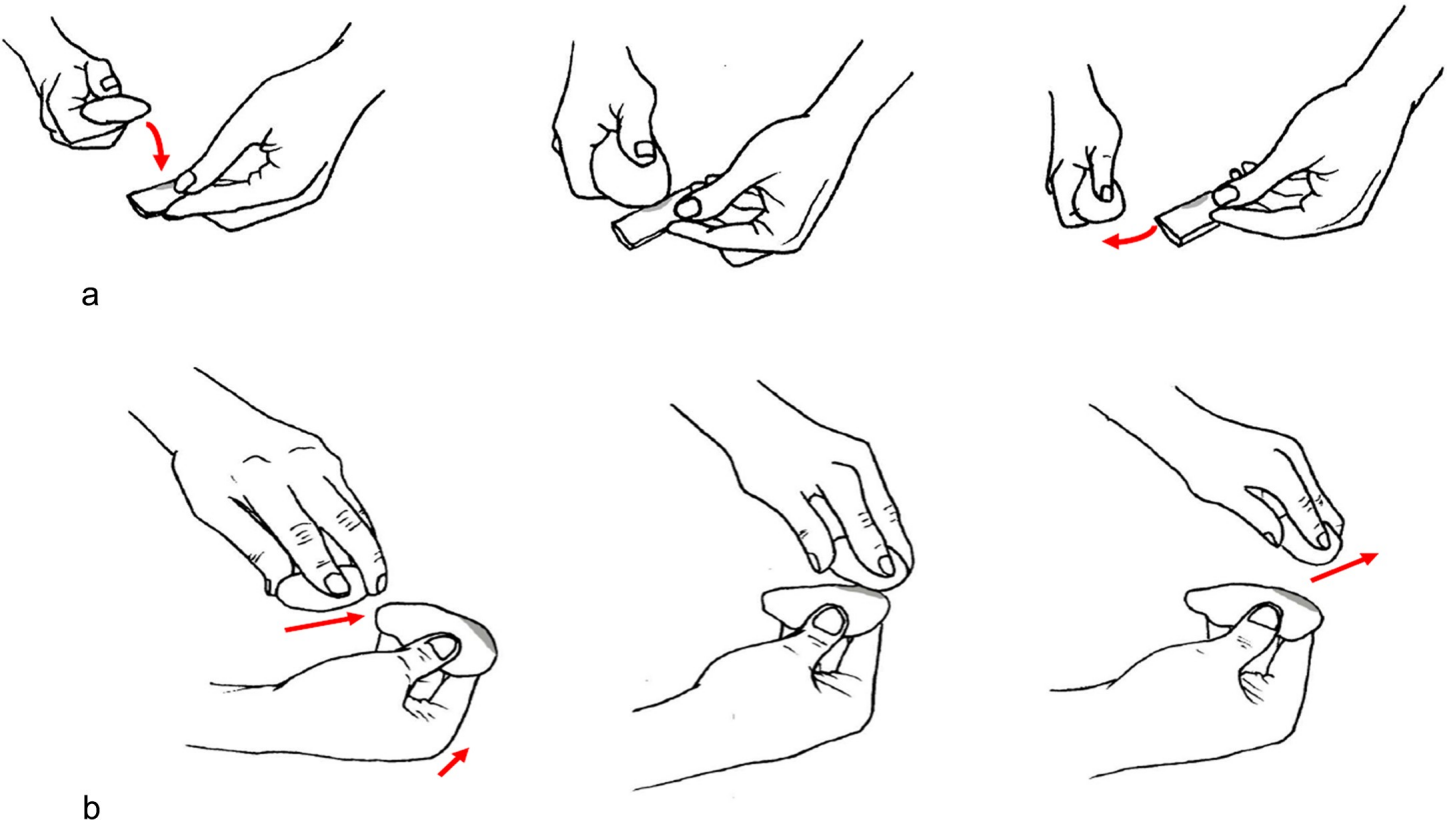
Schematization of the gestures used during the retouching experimental activity. (a) The marginal and abrupt retouch: a rapid and consequential gesture was performed. The knapper’s arm moved following an oblique dragging trajectory against the blank’s edge, striking it very quickly using the tool’s flat face along the apical area; (b) the scaled retouch: this action followed a perpendicular trajectory, with respect to the blank edge, with a movement from the top to the bottom of the arm and a final flexion downwards(drawings by Giulia Formichella).

**Fig 6:**
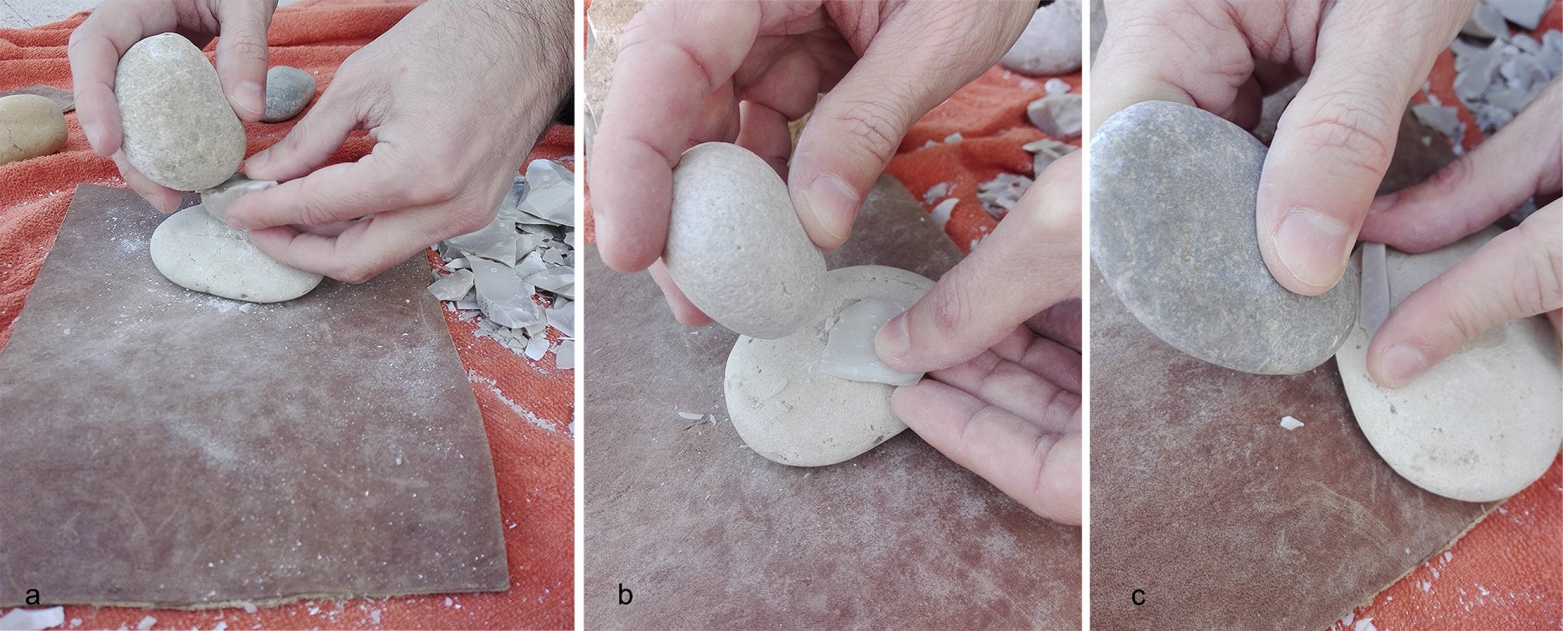
Experimental bipolar percussion and retouch on anvil. (a) Bipolar percussion for flake production; (b) hinged laminar flake retouch on anvil adopting a rectilinear trajectory; (c) bladelet retouch on anvil adopting an oblique trajectory.

Gestures have been described following the criteria outlined by Bourguignon [78]. The following points aim to explain the different phases and the relative technical gestures performed by the expert knapper during the experimental activities:

- cortex removal and core-shaping. The soft stone hammers (103x55mm, average dimensions) were used for opening of the nodules to remove cortical flakes in order to shape a pre-form core composed of a single flaking surface related to a single striking platform. During this step, the hammer’s marginal ends have been used as active parts, performing a punctual gesture consisting of a wide rectilinear trajectory of the arm, related to the force necessary to remove larger products. Despite their effectiveness in flake detachment (cortical and non-cortical), they broke after a reduced number of blows (conchoidal fracture along the functional end or straight fracture following the percussion axis). Therefore, their use during this stage was evaluated as not functional;
- flaking surface and striking platform configuration. After having designed the core volume, the soft hammers (50x50mm, average dimensions) were used to open a flaking surface and prepare the platform and the flaking angle through tiny flake removal. During this phase, flakes of various sizes were removed alternating with abrasion of the overhang performed with the same hammer. This latter action required consequential and rapid gestures with resting percussion, aimed to remove micro-flakes from the overhang. This resulted in a more continuous action that involved a wide contact area–usually along the flat axis or lateral along the pebble edge–between the hammer and the core face. Removals of larger maintenance flakes required slower and more precise blows with a curvilinear trajectory, variable amplitude and force related to the size of the desired flake to be removed. This action involved the use of the marginal ends of the pebble along the minor axis;
- blank production and core maintenance. After having shaped the core, we proceeded to the extraction of lamellar blanks using an organic hammer (deer antler; [120], p.133) and a soft stone hammer, as hypothesized in a recent revision of the bladelets’ technical attributes ([111] p.27). During this phase, the stone hammers (sized 50-40mm in length, average dimensions) were always used with a rectilinear trajectory on their marginal ends. They performed effectively in blade production, even though small conchoidal fractures appeared in the functional area which, however, did not lead to discarding the tool. Flake detachment and abrasion operations were also carried out, aimed at maintaining the flaking angle and the transverse and longitudinal convexities of the core;
- bipolar percussion. Due to the presence of some splintered pieces in the archaeological assemblage ([120], p.139) we tested the bipolar percussion by placing the core on a base, consisting of a large flat pebble selected among the collected items. At this stage, the core was of very reduced size and allowed the application of this technique despite the small size of the anvil (50x50x30 mm, average dimensions);
- retouching. Several gestures have been tested according to the different morphologies and intensity of retouching documented for Protoaurignacian and Aurignacian levels at Fumane cave. The occurrence of retouch features was strictly combined with the gesture and the technique, which involved different uses of the functional areas of the pebbles (e.g. short edge or flat face).
  - Direct percussion. A rapid and consequential gesture was performed: the knapper’s arm moved following an oblique dragging trajectory against the blank’s edge, striking it very quickly using the tool’s flat face along the apical area. This movement allowed the removal of tiny flakes and was particularly effective for delineating straight cutting edges with marginal and abrupt retouch on thinner edges, due to the limited contact area between the hammer and the blank edge of a wide spectrum of blank morphologies from simple flakes to blades sharing a consistent thickness (**Figs 4A and 5B**). We noted that this type of retouch can also be performed with different trajectories (e.g. perpendicular to the blank axis). This technique was also used to delineate the front of carinated end-scrapers and of some thin scrapers, even though the short edge of the retoucher was used. This allowed the removal of tiny bladelets and elongated flakes by adopting a marginal percussion (cfr. [125]). A more punctual gesture produced a more invasive retouch of a scaled type (**Figs 4B and 5A**), due to a larger contact area between the hammer flat face and the blank to be retouched. This action followed a perpendicular trajectory, with respect to the blank edge, with a movement from the top to the bottom of the arm and a final flexion downwards. This type of retouch has been performed on blades for delineating the front of the end-scrapers (cfr. [111,120]).
  - Direct percussion on anvil (**Fig 6**). This technique was aimed at retouching tiny bladelets: a flat pebble was used as anvil on which the blank edge was modified through the use of a retoucher by percussion ([98,126,127]). The trajectory was found to be variable depending on the position of the blank to be retouched on the anvil: in a central position a perpendicular trajectory was adopted, while when slightly inclined in proximity of the lateral edge of the anvil an oblique trajectory was adopted. In both cases, the short edges of the retoucher were used.
  - Edge abrasion (or *égrisage*, [127]). The bladelet edge was modified by rubbing against the pebble with the aim of delineating a straight edge (**Fig 4C**). This reciprocal contact permitted the detachment of micro-flakes.

## Results

The replicas used during the experimental protocol comprised: a) hammerstones, used for removing cortex and shaping cores, abrasion of core edges and detachment of flakes and bladelets (n.9); b) retouchers, used to produce different types of retouch (n.5); c) anvil, used as a passive base for detaching flakes (n.2); hammerstones/retouchers used with mixed activity (n.3).

### Hammerstones

**Fig 7:**
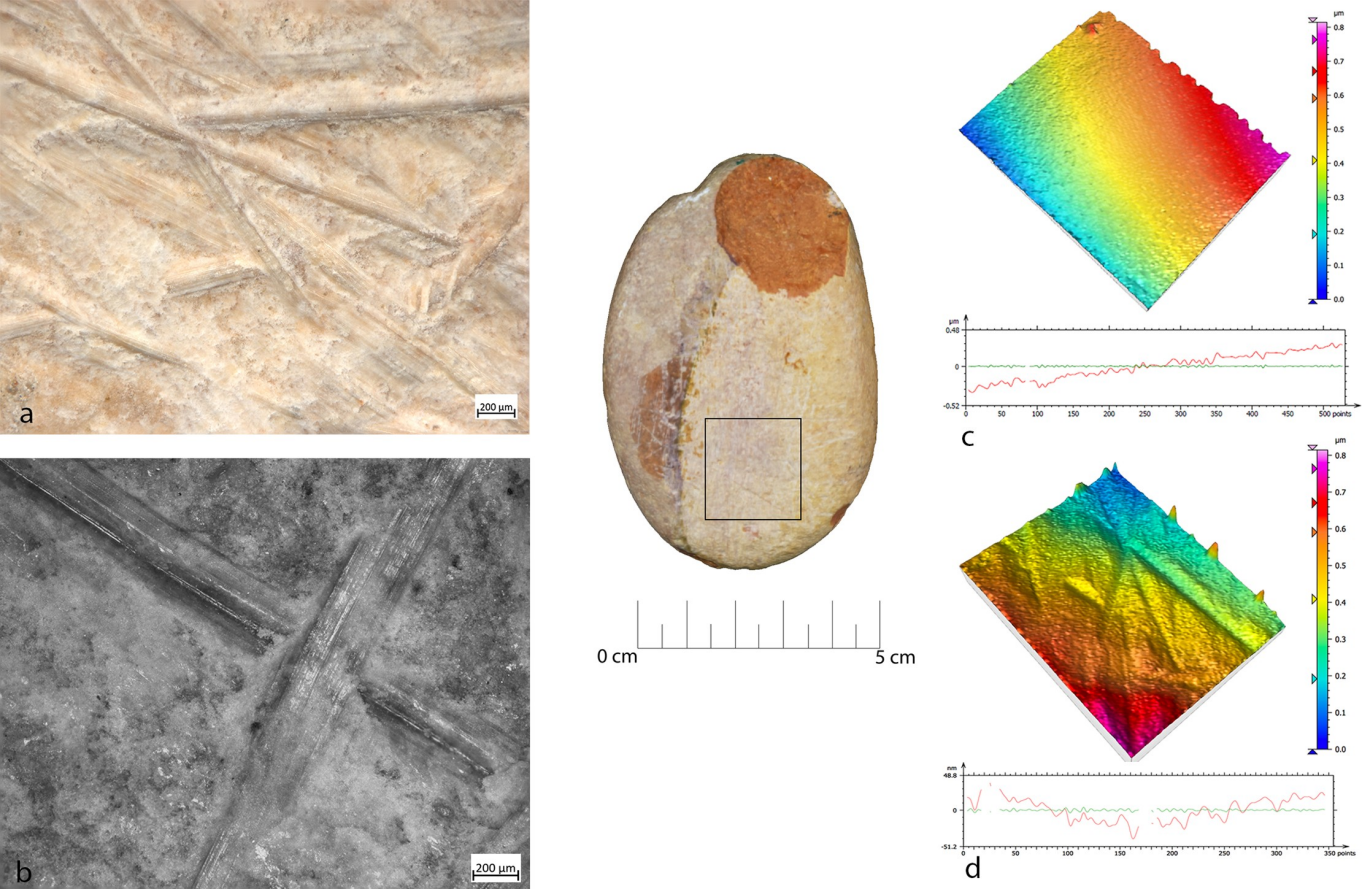
FSPM-13 use wear on experimental replica used in overhang abrasion. (a) Macro-traces (30x) long, deep striations alternate with more superficial striations, with different orientations. They are located on the flat and/or on the long edge of the instrument; (b) micro-traces (200x), striations with polishes on the bottom, with rough texture; (c) 3D microtopography of the unused surface and profile; (d) 3D microtopography of the used surface and profile.

**Fig 8:**
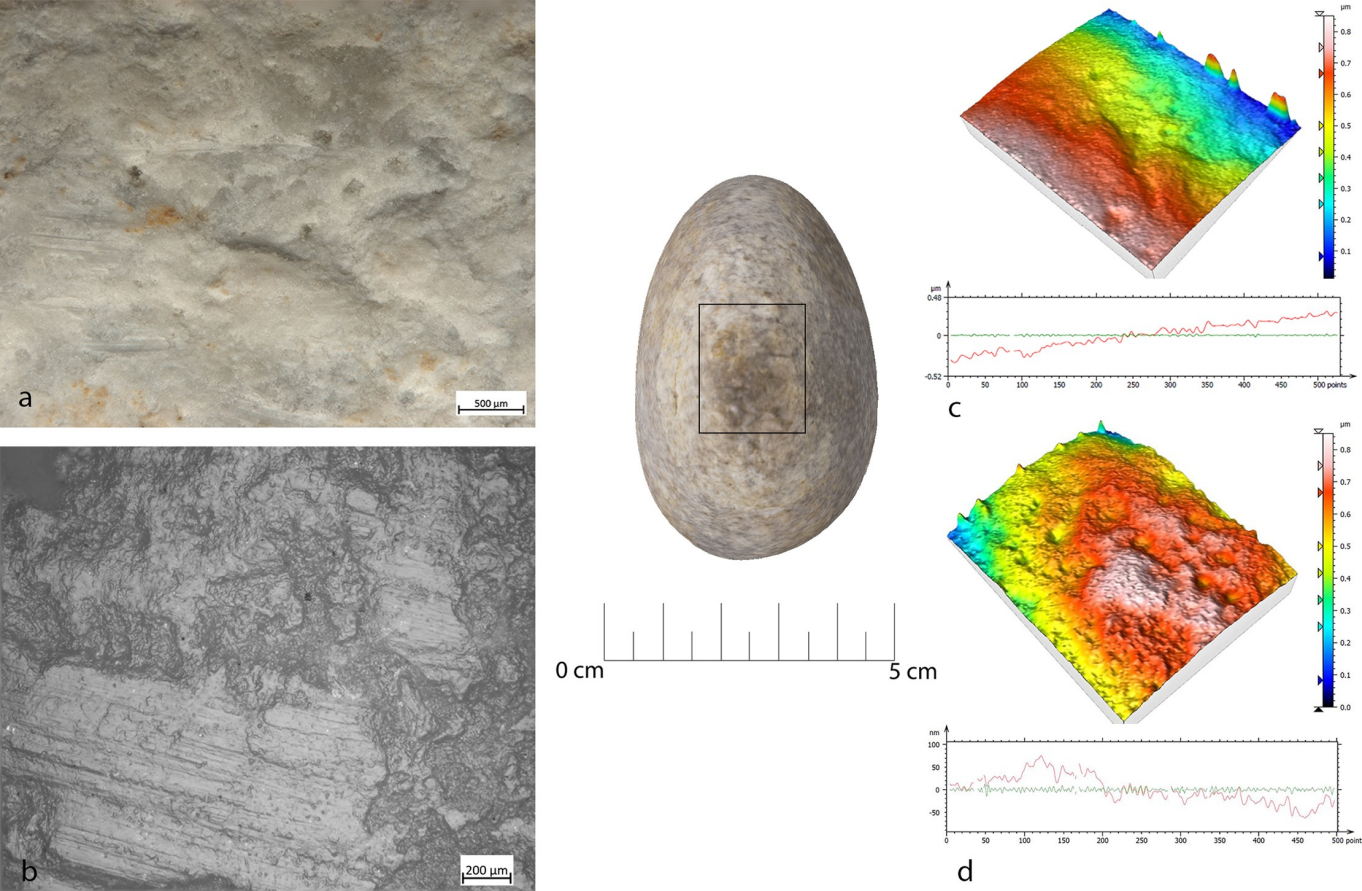
FBR-2 use wear on an experimental replica used in the configuration of the striking platform of the core. (a) Macro-traces (30x) highlight the presence of overlapping pits with sub-oval morphology, located all around the marginal perimeter of the object; (b) micro-traces (200x) are extended onto the top of the grains, with smooth texture, flat topography, striation with the same orientation, and concentrated-separated distribution; (c) 3D microtopography of the unused surface and profile; (d) 3D microtopography of the used surface and profile.

The types of use-wear observed on the hammerstones were:

- during cortex removal and core-shaping large longitudinal flake scars (50mm) located along the short edge were produced. In association with these scars there were residual surfaces with pits, similar to the deep scales, of around 6mm in size, with a triangular section. Micro-polishes were absent.
- Overhang abrasion activity produced long, deep striations alternated with more superficial striations, with different orientations, often associated with the configuration of the striking platform. These striations were located on the flat and/or on the long edge of the tool and showed polishing on the bottom with a rough texture when observed at the metallographic microscope (**Fig 7**).

- During the configuration of the striking platform of the core, the removal of small flakes produced small pits with sub-oval morphology. The pits often overlapped short superficial striation. These traces were located on the short edges of the tool; if this is circular, use-wear traces were distributed all around its perimeter. A micro-polish was observed, extended on the top of the grains, with a smooth texture, flat topography, uniformly oriented striations, with concentrated-separated distribution (**Fig 8**).

- During blank production and core maintenance small sub-circular pits overlapping with small striations and chaotic orientation were produced; flake scars (20/30mm) due to the blow for the extraction of the blank were also observed. The mechanical levelling led to the production of short strips and sporadic polishing with loose-separated distribution on the top of the grains, with deep striation with the same orientation, and a rough texture and domed topography. The traces were located on the short edge of the hammerstone.

Pits produced by the trimming of the striking platform and the production of blanks and core maintenance looked very similar in their distribution and morphology. Often overlapping, pits were not well defined, but polishes looked different. In particular, the trimming of the striking platform produced polishing as a consequence of repeated contact between the hammer and the edge of the flint tool. On the contrary, the detachment of the blades/bladelets consisted of a more precise blow.

### Bipolar percussion

**Fig 9:**
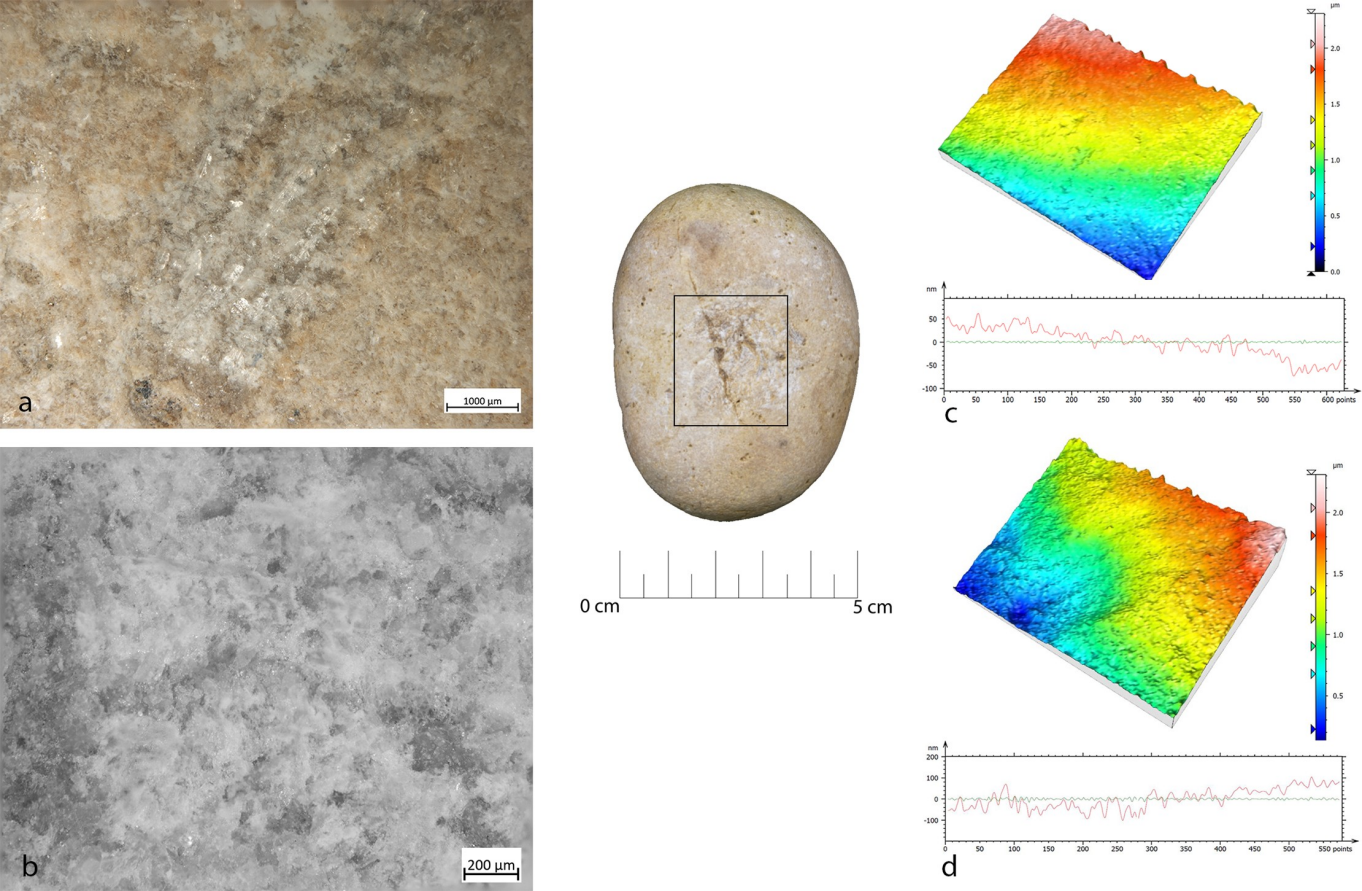
FA-8 use wear on experimental replica used in passive percussion. (a) Macro-traces (40x) consisted of large pits with sub-quadrangular/triangular morphology, grains appeared fractured, located in the central area of the flat surface of the pebble; (b) the micro-traces (200x) are absent, the bottom of the pits appear rough; (c) 3D microtopography of the unused surface and profile; (d) 3D microtopography of the used surface and profile.

Bipolar percussion makes large pits with sub-quadrangular/triangular morphology located in the central area of the flat surface of the tool. The texture grains appear fractured, polishes are absent (**Fig 9**).

## Retouchers

**Fig 10:**
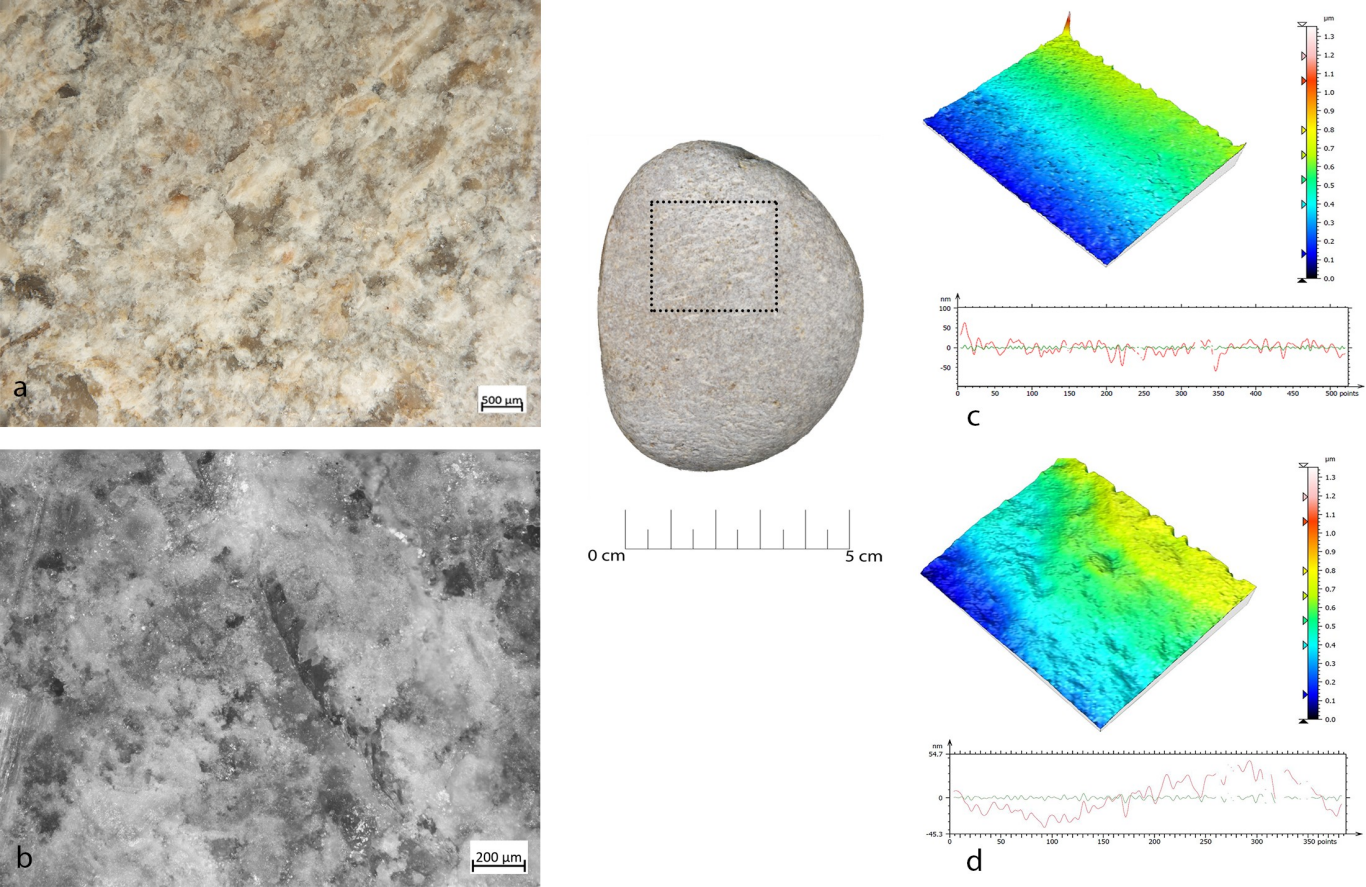
FRS-1 use wear on experimental replica used in scaled retouch. (a) Macro-traces (30x), contiguous pits of linear form (half-moon), with rough bottom, and triangular section; the traces are located in the centre of the apical area; (b) polishes (200x) are absent; (c) 3D microtopography of the unused surface and profile; (d) 3D microtopography of the used surface and profile.

Retouchers presented different types of use-wear. In detail, the scaled retouch generated a series of contiguous pits of a linear form (reduced half-moon) with a rough bottom and an asymmetric triangular section, localised on the flat surfaces of the tool concentrated near the apices. There were also striations: short, more sporadic and superficial (**Fig 10**). The micro-polishes were probably absent because the traces resulted from a punctual contact between the retoucher and the edge of the flint tool (the dragged gesture is absent).

**Fig 11:**
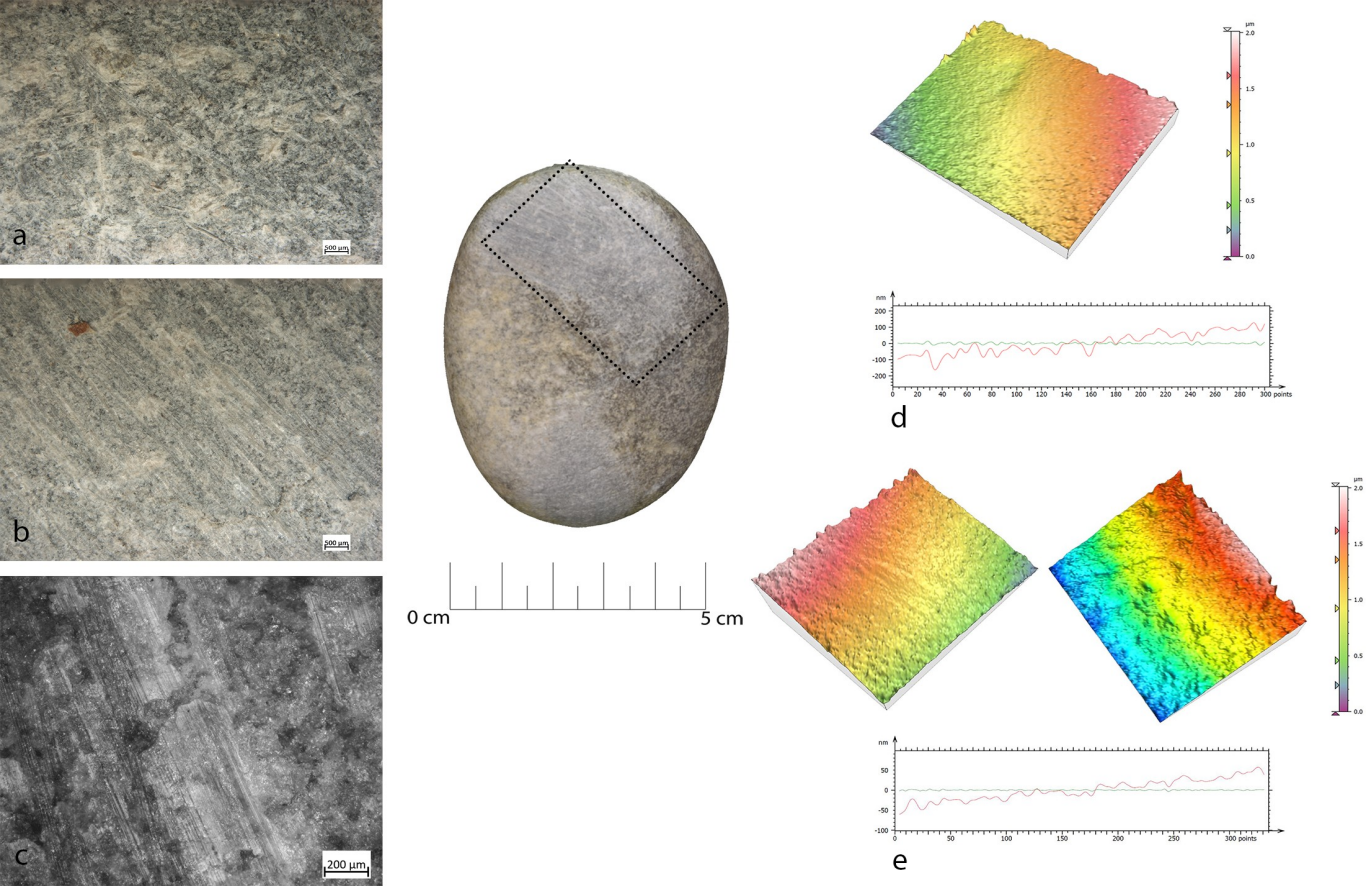
FRP-1 use wear on experimental replica used in marginal retouch. (a) Macro-traces highlight area characterized by a concentration of micro-pits (25x) with sub-circular morphology; (b) long striation (20x) associated with the pits and with the same orientations; the traces are located in apical top with oblique orientation; (c) micro-traces (200x), band of polishes with striations, covered-closed distribution, rough texture and domed topography; (d) 3D microtopography of the unused surface and profile; (e) 3D microtopography of the used surface and profile (striations and pits).

Marginal retouch led to an association of small circular pits and dense long parallel striations. Use-wear traces concentrated over the apical area of the flat surface, with oblique orientations. Bands of polishing with striations were present, with covered-closed distribution, a rough texture and domed topography. The dragging movement (oblique trajectory) related to the marginal retouch, produced a mechanical levelling of the surface where the polishes were present (**Fig 11).**

Other types of retouching were tested, including edge abrasion. This activity produced traces located in a small area between the short edge and the apical area of the retoucher. The traces consisted of small pits with sub-quadrangular morphology and short striations. The rough polishes were present.

**Fig 12:**
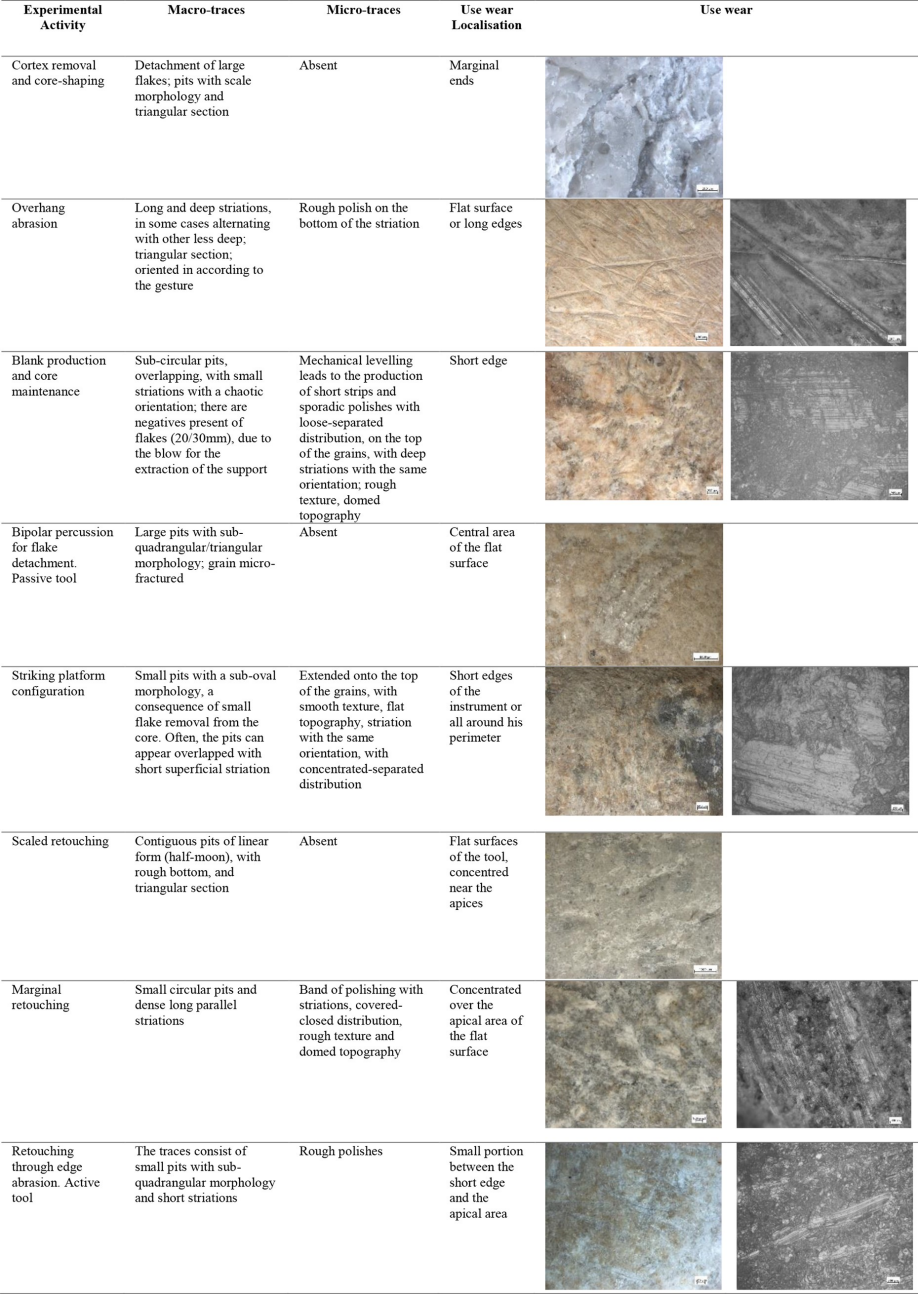
List of experimental activity and use wear associated. Description of activities, macro and micro traces, use wear localisation and pictures.

Retouching on an anvil produced, on the passive base, superficial small pits with a sub-circular morphology and short striation. The use wear was located on the flat surface. The polishes were absent. The same traces were present on the active retoucher but located on the short edge (**Fig 12**).

**Fig 13:**
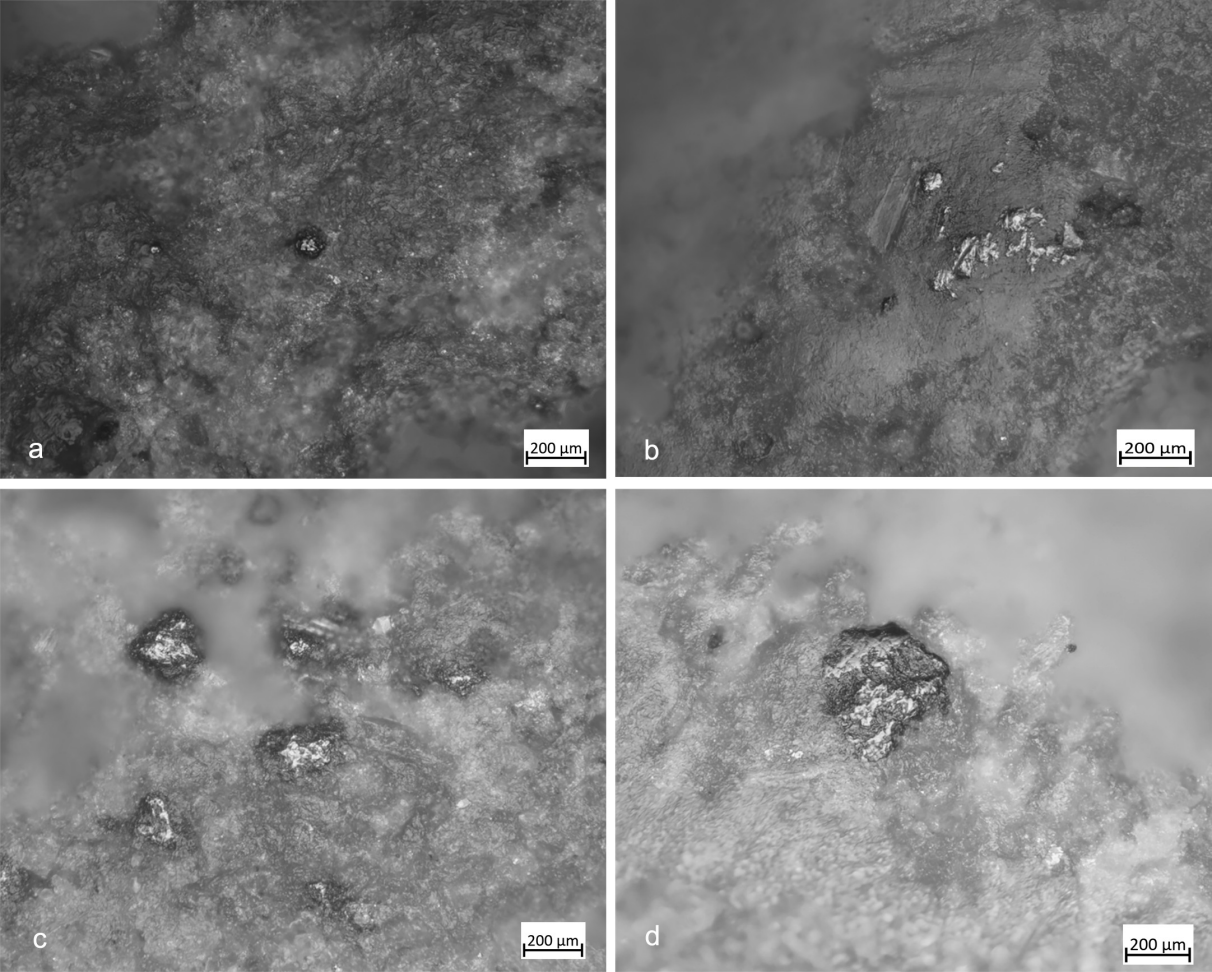
Experimental use wear related to the prehension. (a-b) Patch of polishing visible through the metallographic microscope (100x), localised on the top of the grain; (c-d) smooth/flat patch of polishing visible through the metallographic microscope (200x).

On the experimental samples, prehension traces were visible at high magnification. Macroscopically, the prehensive area was smoothed, with several patches of smooth/flat polishing, affecting the top of the grains. Polishes visible between 20x and 50x developed on the flat and central portion of the tool, favoured by a type of prehension in which a large portion of the finger (fingertip) was in contact with the flat surface of the tool. Polishing was not observed in cases where the hammer or the retoucher was gripped by the short margins (tridigital prehensions) and the contact occurred with a reduced portion of the finger (**Fig 13**).

### GIS analysis—Experimental sample

**Fig 14:**
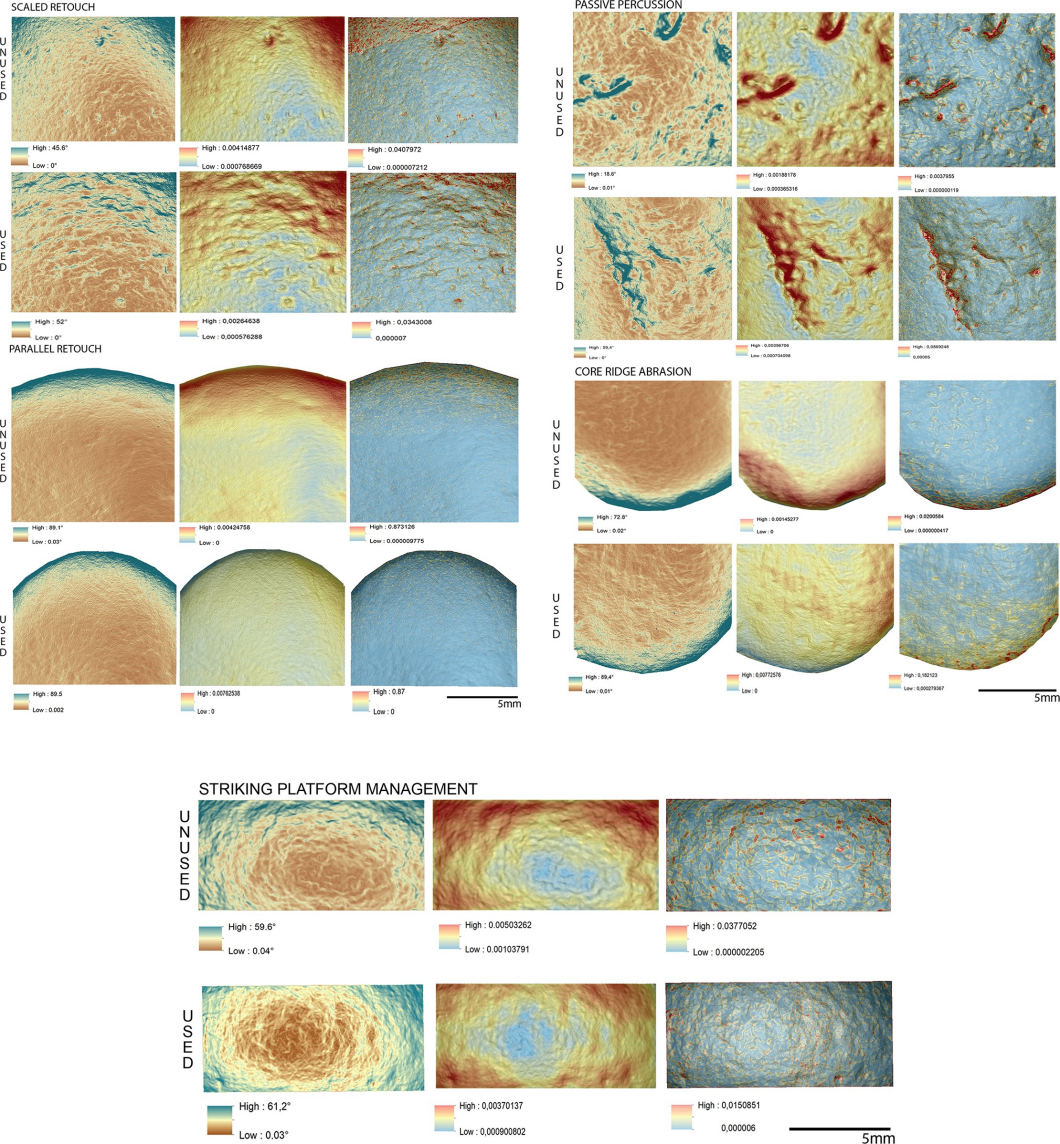
Comparison of the surface topography before and after the use of the tool to produce scaled and marginal retouch, percussion activities and bladelet production. Digital Surface Maps of Slope, TRI and VRM respectively.

Overall, the raw material characterising the experimental sample presented in this work was homogeneous. This led to minimal modifications of the tools surfaces in particular concerning their roughness. On the other hand, the analysis of slope revealed several differences between the activities performed (**Fig 14**).

**Fig 15:**
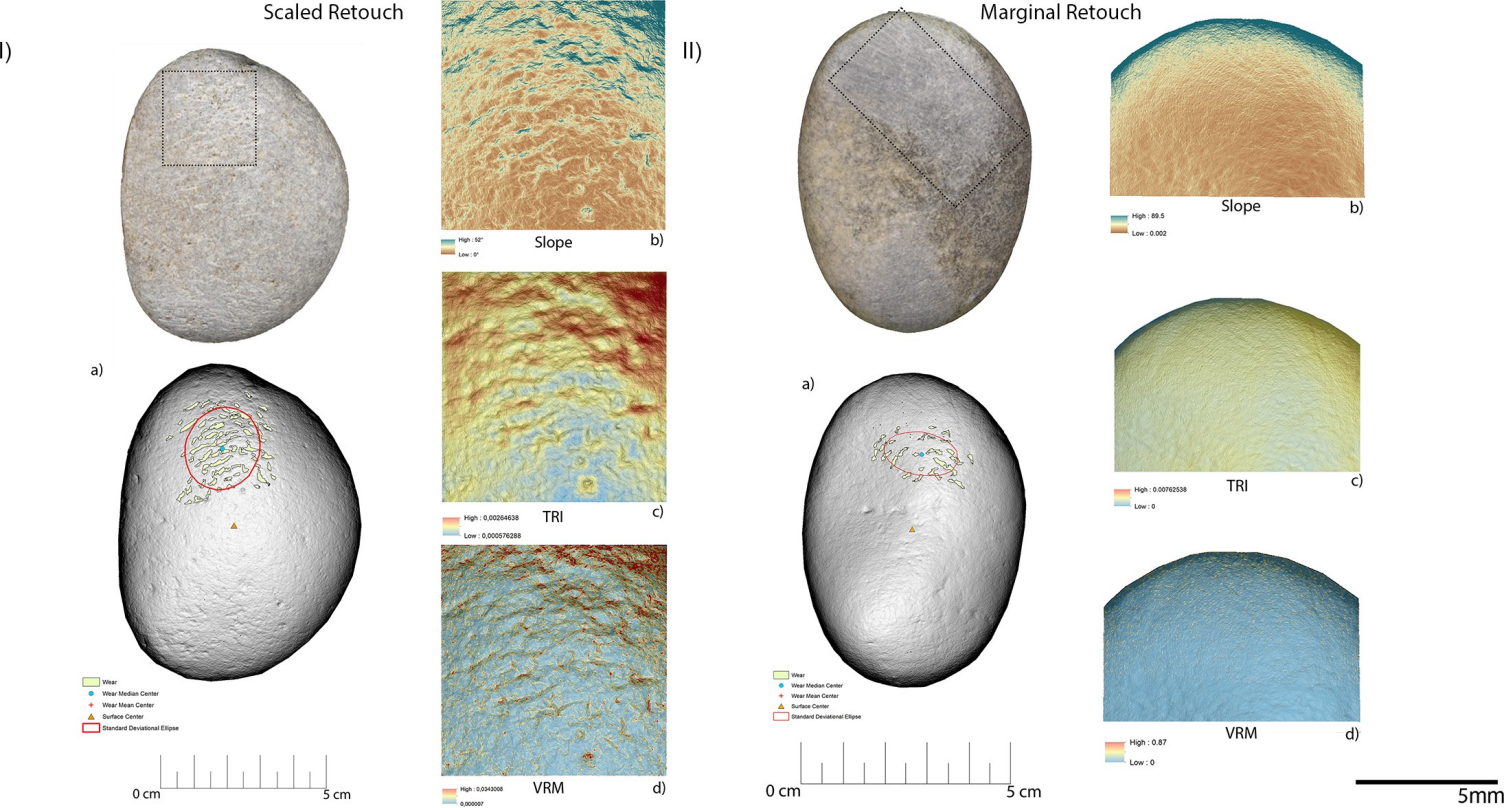
Experimental objects utilized for scaled retouch (I) and marginal retouch (II). (a) Spatial distribution of the identified wear; (b) slope; (c) terrain roughness index; (d) vector roughness measure.

The experimental replicas utilised to produce scaled retouch recorded the development of depressions exhibiting a mean slope value of 9.94°. Surface ruggedness measured through TRI and VRM appeared low with a mean TRI value of (0.0015) and a VRM mean value of (0.001). Most of the variability was concentrated over the apices of the tool. Scaled retouch (**Fig 15I, Table 3**) led to the development of use-related wear on the apical portion of the tool. Wear features were characterised by an average perimeter and an area of 8.6 mm and 1.5 mm^2^ respectively. The average distance of the wear feature from the centre of the tool was 16 mm while the average from its edge was 13 mm. Traces are concentrated over the central portion of the tool apex as suggested by the standard deviational ellipse elongation value (0.87 ad). As in the case of scaled retouch, use wear generated by marginal retouch (**Fig 15II, Table 3**) also affected the apical portion of the retoucher. The depression caused by use featured a slope mean value of 18.8°. The surface showed an overall homogeneity as indicated by the recorded TRI (0.0015) and VRM (0.0023) mean values, with most of the surface variability localised on the tool apical areas. Use related wear exhibited an average perimeter of 9 mm and a mean area of 1 mm^2^ along with an average distance from the tool centre and edge of 15 mm and 12 mm respectively. Traces generated by marginal retouch were well spread over the retoucher apical portion as suggested by the standard deviational ellipse elongation value (1.7 ad), higher than the value observed on the experimental replica used in scaled retouching.

**Table 3:** Morphometric features of the wear identified on the utilised areas of the experimental replicas. In detail, scaled retouch (FSR-1), marginal retouch (FRP-1), bipolar percussion (FA-8), striking platform maintenance (FSPM-13) and bladelet removal (FBR-2).

	FRS-1	FRP-1	FA-8	FSPM-13	FBR-2
**Perimeter (mm)**					
*Minimum*	3.5	3.2	3	2.9	1.6
*Maximum*	34	21	28	74	22
*Average*	8.6	9	7.2	14	3.8
**Area (mm2)**					
*Minimum*	0.5	1	1	1	1
*Maximum*	6.7	5	9	17	5
*Average*	1.5	1	1	3	1
**Distance from Centre (mm)**					
*Minimum*	7.6	9.7	0.1	11	
*Maximum*	26	21	18	33	
*Average*	16	15	9	20	
**Distance from Edge (mm)**					
Minimum	4.1	8.1	12	1.8	
Maximum	21	18	27	20	
Average	12	12	21	10	
**Standard Deviational Ellipse**					
*Perimeter (mm)*	52	38.8	61	63	43.2
*Area (mm2)*	216	106	281	311	116
*Elongation (ad)*	0.87	1.7	0.7	1.2	2.3
**Used Area (%)**	3	1	3	6	2
**Pits Density (mm2)**	0.02	0.01	0.02	0.02	0.02

**Fig 16:**
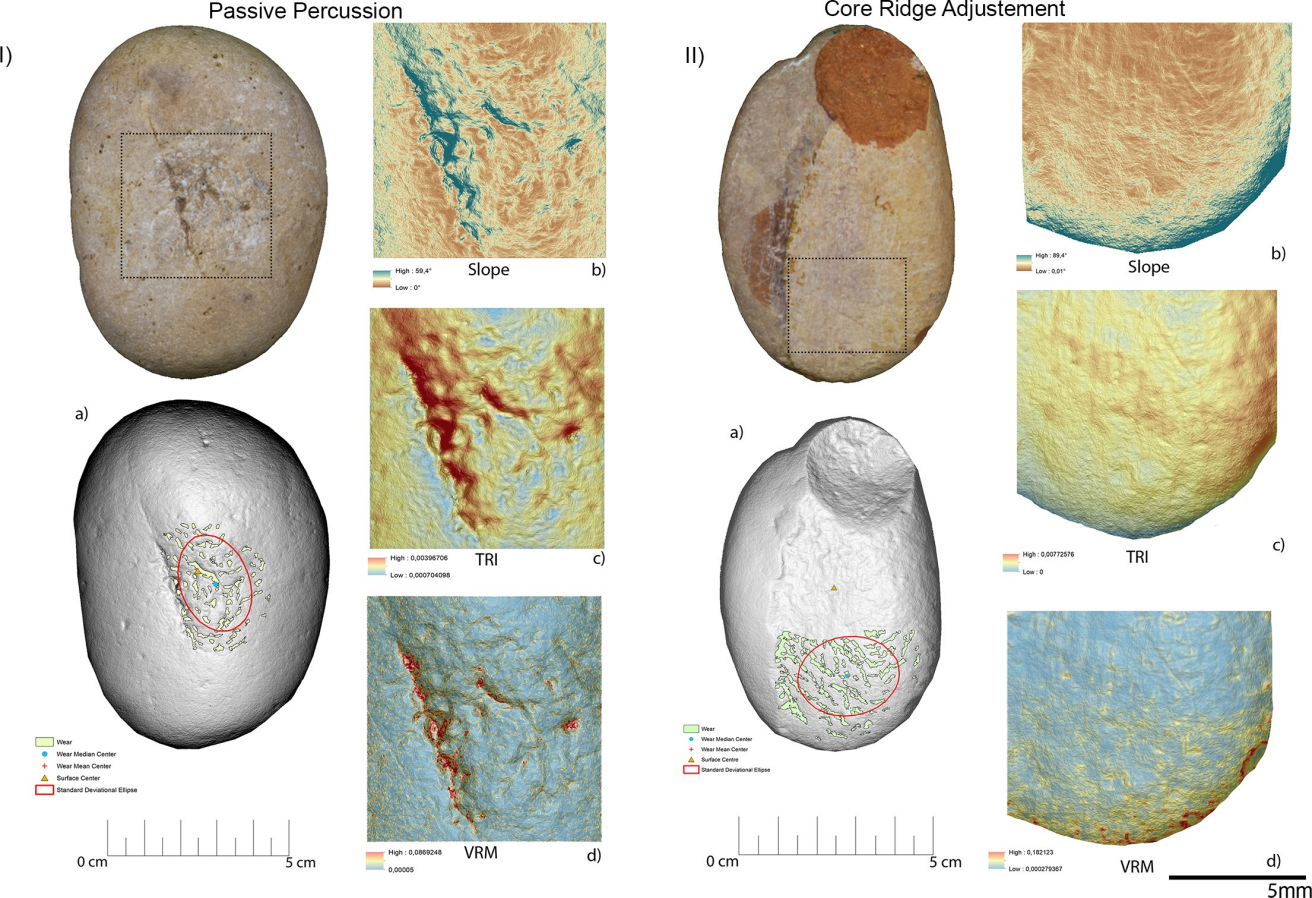
Experimental object utilized in passive percussion (I) and core ridge adjustment (II). (a) Spatial distribution of the identified wear; (b) slope; (c) terrain roughness index; (d) vector roughness measure.

Passive percussion (**Fig 16I, Table 3**) led to the development of wear over the central area of the tool used as anvil, where depressions developed featuring a mean slope value of 11.2°. The surface was overall homogeneous (TRI mean value 0.007) with a low topographic variability (VRM mean value 0,0009) mostly at the bottom of the produced wear. Use marks generated by passive percussion featured a mean perimeter of 7.2 mm and an average area of 1 mm^2^. Traces were localised near the centre of the tool, with an average distance from the centre of 9 mm, while their average distance from the edges averaged 21mm. Traces were concentrated on the tool surface centre as indicated by the standard deviational ellipse elongation value of 0.7 ad.

Adjustment of core ridges (**Fig 16II, Table 3**) led to the development of use wear over the apical portion of the tool and in a minimal part over its inner areas. Depressions caused by use featured a mean slope value of 14°, while the TRI and VRM mean values, 0.0011 and 0.0034 respectively, suggest an overall homogeneous surface topography with its higher topographic variability localised over the outer portion of the tool apical area. Marks generated by use were relatively large given their average perimeter of 14 mm and mean area of 3 mm^2^. While the inner area of the object was also affected, most of the traces generated by the adjustment of core ridges were located near the tool edge (average distance 10 mm) rather than its centre (mean distance 20 mm). Use related damage was well spread over the affected area of the tool as indicated by the standard deviational ellipse elongation value of 1.2 ad.

**Fig 17:**
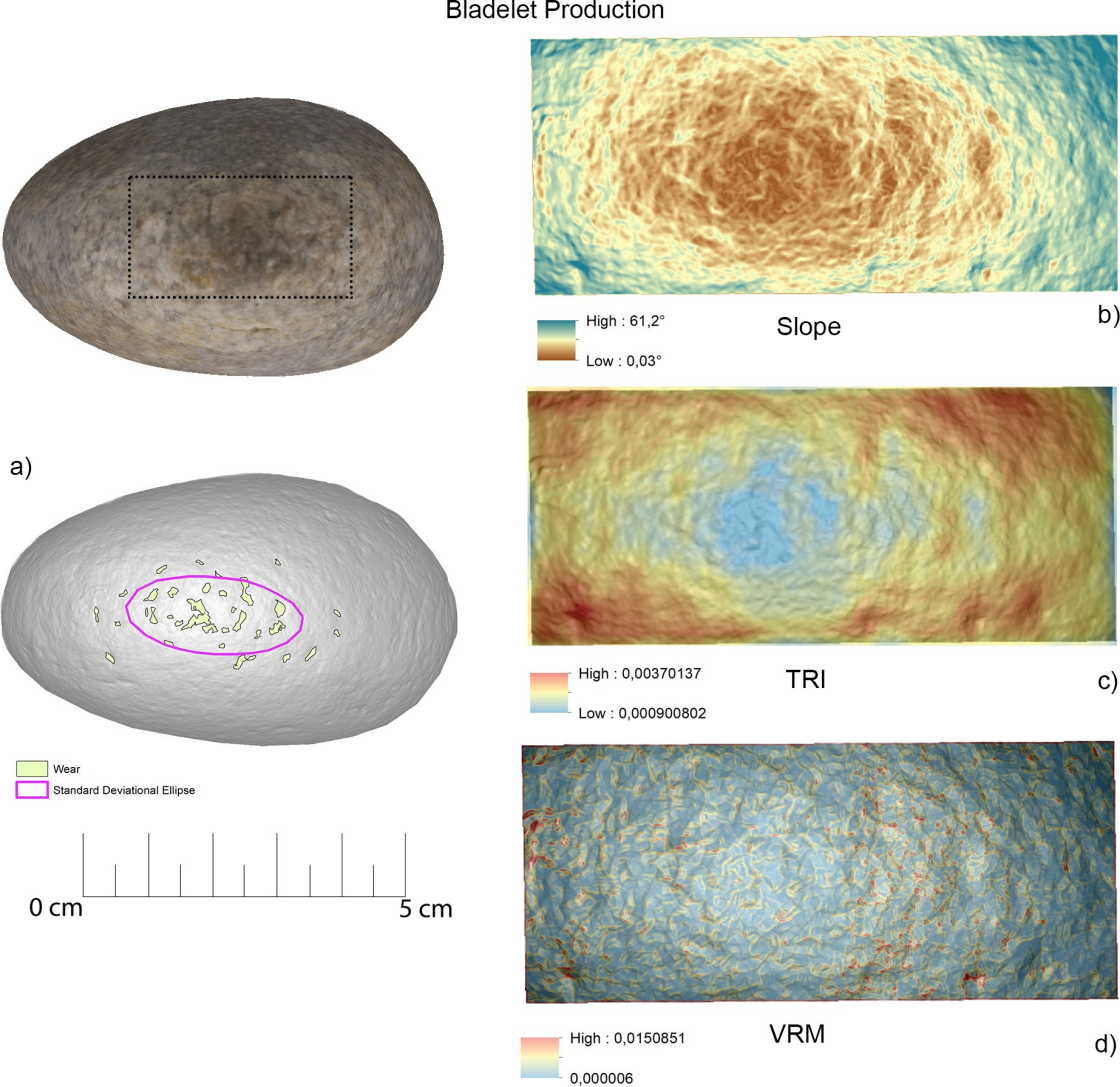
Experimental object utilized for bladelet production. (a) Spatial distribution of the identified wear; (b) slope; (c) terrain roughness index; (d) vector roughness measure.

**Fig 18:**
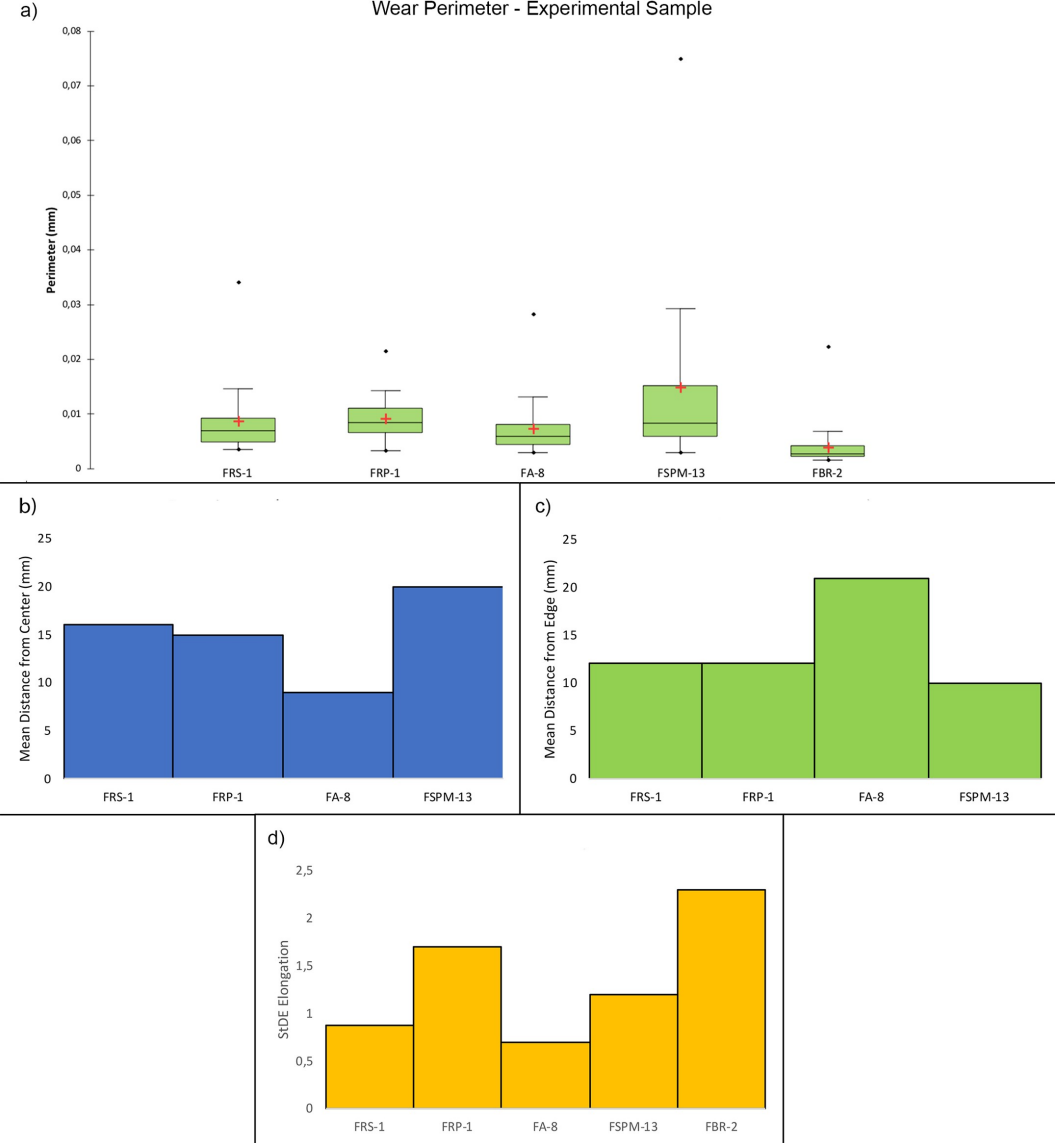
Perimeter of the wear. (a) Dimensions of the wear identified over the utilized areas of the experimental replicas; (b) mean distance of the identified wear from the object centre; (c) mean distance of the identified wear from the object edge; (d) dispersion of the identified wear over the tool surface defined by the elongation of the standard deviational ellipse.

For the purpose of bladelets production (**Fig 17, Table 3**), the short edge of the experimental replicas was used rather than its surface. Over the used portion the depressions generated by use were characterised by an average slope value of 23.7°. The used area of the tool was characterised by a higher degree of heterogeneity when compared to the other experimental samples presented in this work, as indicated by TRI (mean value 0,0025) and VRM (mean value 0,0067). Of particular interest is the fact that surface roughness was lower in proximity to the centre of the used surface area, where the bigger traces were located. Wear generated by bladelets production featured an average perimeter of 3.8 mm and a mean area of 1 mm^2^. Damage affected most of the used area of the tool as indicated by the standard deviational ellipse elongation value (2.3ad) (**Fig 18, Table 3**).

### Archaeological sample

#### Use wear analysis

In the archaeological sample, traces of use were identified on 7 objects. These allowed the determination of the use of the tools at Fumane cave as: **a)** hammerstones (n. 3, RF37, RF138, RF73); **b)** retouchers (n.2, RF80 and RF67); **c)** hammerstone/retoucher (n.1, RF127); anvil/retoucher (n.1, RF92). The artefacts showed a general rounding due to post-depositional alteration, probably of chemical nature. Invasive patinas or concretions were visible in one case (RF73 around the edge) (**Table 4**).

**Table 4:** Archaeological sample and use wear description and interpretation.

Id	Type	Activity	Macro traces	Micro traces	Traces Localisation	Prehension	Note
RF73	Hammerstone	Core maintenance and overhang abrasion	Isolated striations, chaotic, deep and long; overlapping pits.	Absent	Pits located on short margins; striations on the flat surfaces	Absent	The sample is altered (grain detachment and rounding)
RF127	Hammerstone and Retoucher	Core maintenance and scaled retouch	Small pits, overlapping with associated small striations; linear (half-moon) pits.	The bottom of linear pits is not polished	Pits overlapping located all around the short margin; linear pits located on the two flat surfaces	Yes, in the central area, on the flat surface (rounding of grain, organic film, and patches of polish)	
RF67	Retoucher	Scaled retouch	Linear (half-moon) pits	The bottom of linear pits is not polished	Pits located on the two flat surfaces, opposite apices	Absent	Ochre residues; General rounding
RF92	Anvil and retoucher	Marginal retouch; passive anvil	Long, superficial striations with the same orientation, associated with small sub-circular pits; pits with sub-triangular or quadrangular morphology.	The bottom of the striations is not polished	Pits and associated striations located along the apices of the flat surfaces; pits with sub-triangular/quadrangular morphology in the central area on the flat surface	Absent	
RF80	Retoucher	Marginal retouch	Circular pits and associated striations on the apical areas of the flat surface	The bottom of the striations is not polished	On the apical areas of one flat surface	Absent	
RF138	Hammerstone	Bladelet removal	Flake detachment and overlapping pits. Morphology of the pits is not defined.	Polishing not present	Along the short, opposing, edges	Absent	Alterations, general rounding
RF37	Hammerstone	Overhang abrasion and percussion activity	There are long and deep striations and overlapping pits	Absent	Striations on one of the flat surfaces; and sporadic pits on a long margin	Absent	Alteration due to thermal contact; general rounding

#### Hammerstones

**Fig 19:**
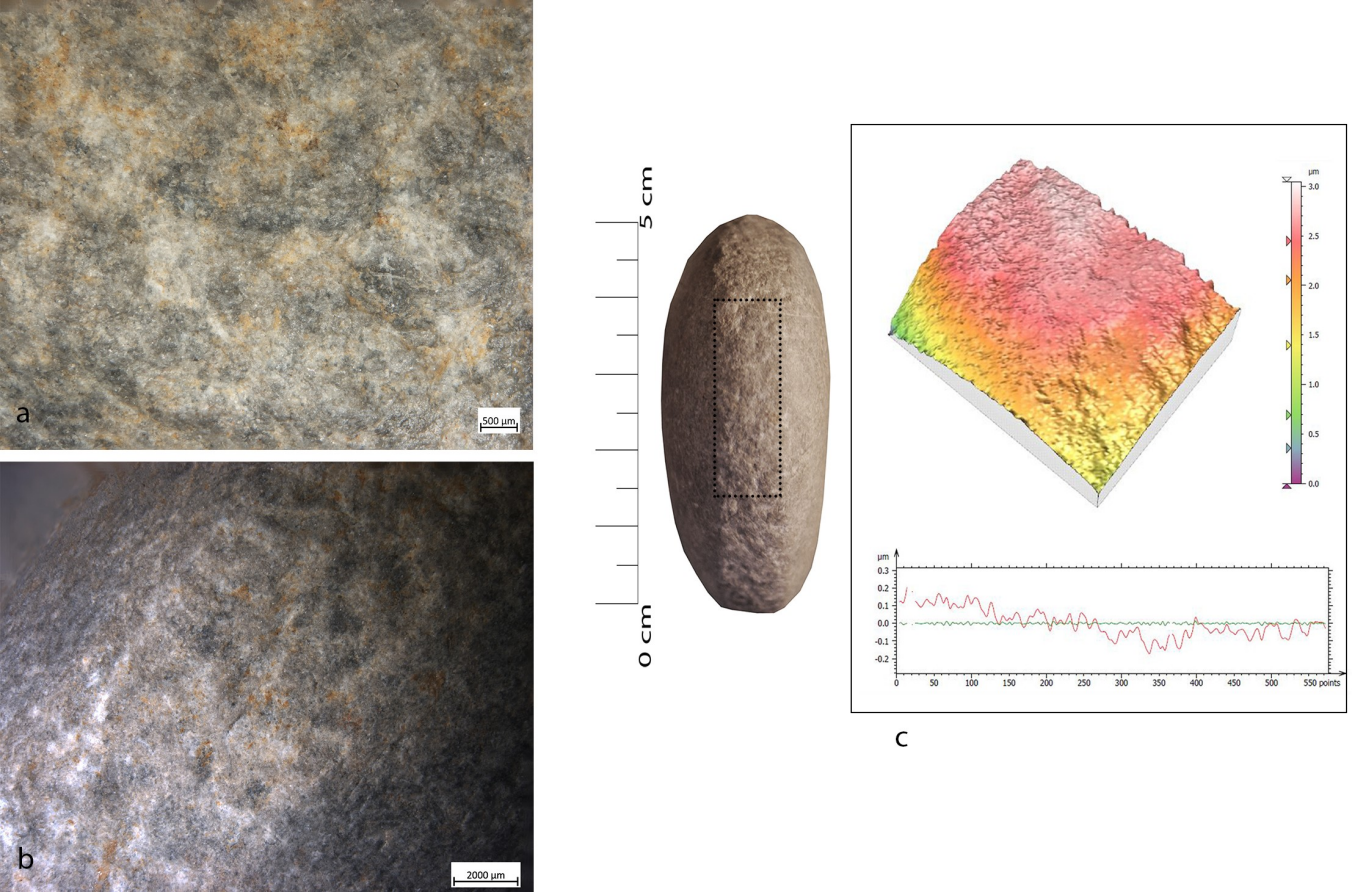
Use wear identified on artefact RF127. (a) Macro-traces (25x), overlaid pits with sub-circular morphology; (b) macro-traces (10x), pits located around the short edge of the artefact; (c) 3D microtopography of the used surface and profile.

**Fig 20:**
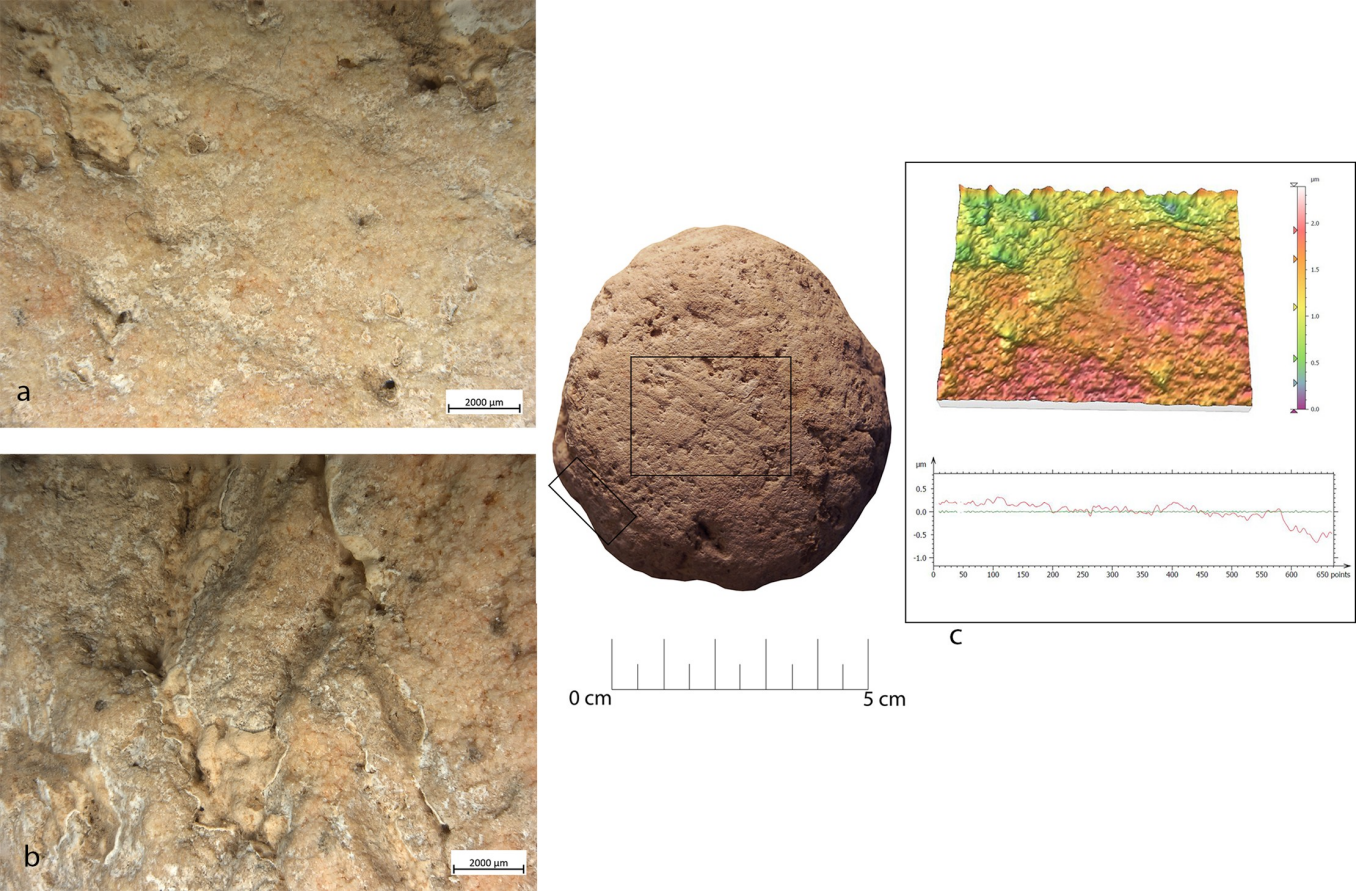
Use wear identified on artefact RF73. (a) Macro-traces (20x), long striations with different orientations located on the flat surfaces in the central area; (b) pits (20x) on the marginal surface, overlapping, covered by the patina. The artefact is affected by dissolution; (c) 3D microtopography of the used surface and profile.

**Fig 21:**
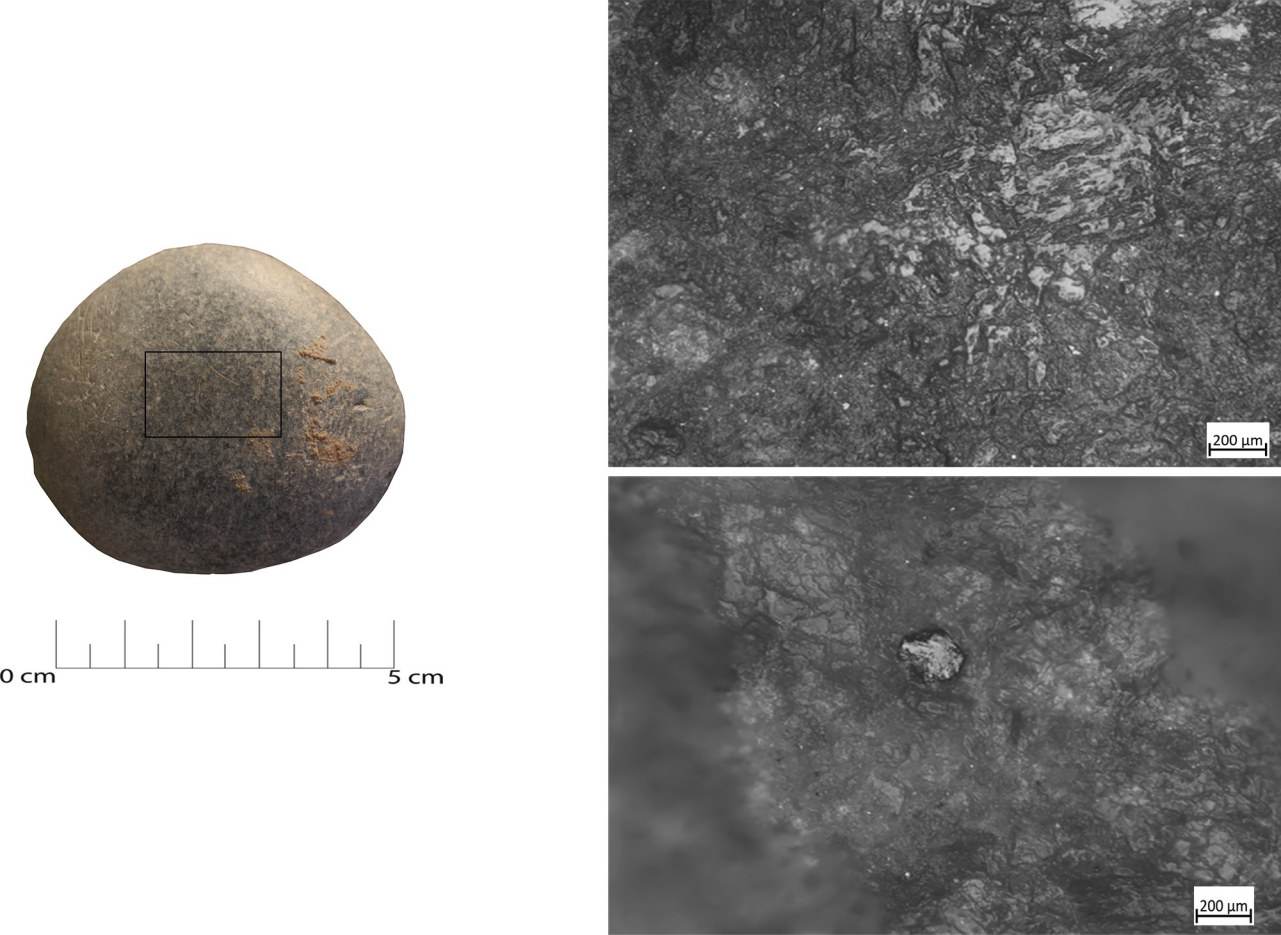
RF127b archaeological sample with intense rounding of the grains over the central area of the flat surface. (a) Polishing (100x) affecting the top of the grains; (b) patch of polish characterised by a flat topography and smooth texture (200x).

In four cases (RF127, RF73, RF138, RF37) pits and flake scars were localized on the short edges of the tool, namely on the opposing short margins or, if the instrument presents a sub-circular shape, all around its perimeter. Small pits overlapped, often associated with short and chaotic striations (RF127) (**Fig 19**). Polishing was not present, probably due to the overall rounding of the surface caused by post-depositional alterations. For the same reason, the pits morphologies were not well defined. However, they shared characteristics similar to those observed on wear produced during core maintenance, related to the detachment of small flakes observed during the experimental knapping of bladelets. One hammerstone (RF138) was characterised by pits associated with negative flake scars (average dimensions 25mm) localised on the short edge of the tool. Deep, long striations were localised on the flat surface, or on the long edge (RF73 and RF37). The flake scars looked very similar to the experimental ones produced during bladelet removal and in overhang abrasions during core management. In one case (RF73) there was an association between the pits, in the marginal extremities, and long and deep striations on the flat surface (**Fig 20**). Moreover, on RF127 polishing was observed associated with intense rounding of the grains over the central area of the flat surface. These latter patches of polish, affecting the top of the grains were characterised by a flat topography and a smooth texture similar to that observed on the experimental sample and related to prehension (**Fig 21**).

#### Anvil

**Fig 22:**
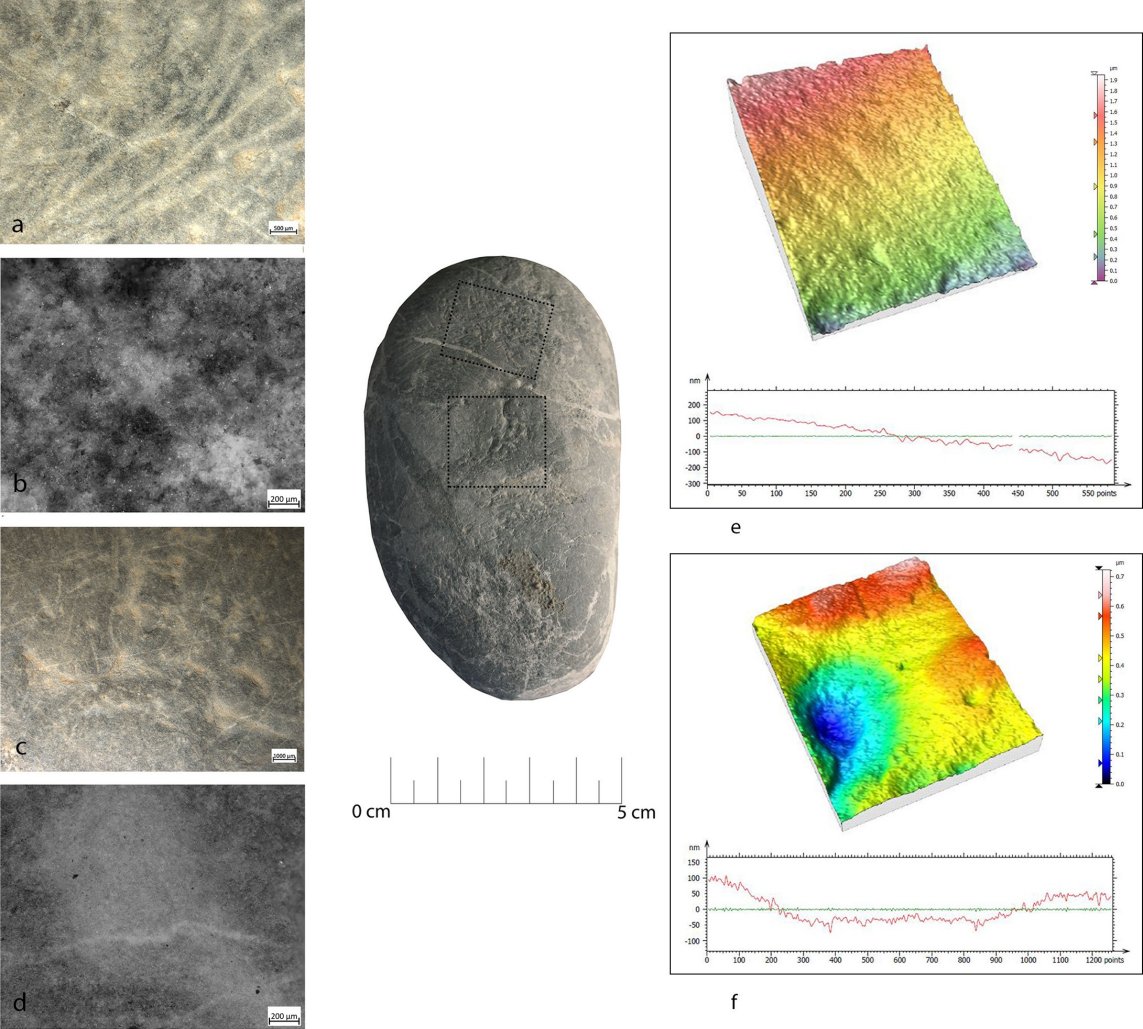
Use wear identified on artefact RF92. (a) Macro-traces (20x), micro-pits with sub-circular morphology associated with long parallel striations, located in the apical top with oblique orientation; (b) micro-traces (200x) are absent, a general rounding is visible; (c) macro-traces (20x), pits with triangular morphology, located in the centre area of the flat surface; (d) polishing is absent (200x); (e) 3D microtopography and profile of the used surface related to (a-b); (f) microtopography and profile of the used surface related to (a-b).

Artefact RF92 featured pits with sub-triangular morphology over its central area. These had a rough bottom with microcracks visible over the grains. Polishing was not present. The observed functional patterns were similar to the experimental sample used as a passive anvil for flake detachment (**Fig 22**).

#### Retouchers

**Fig 23:**
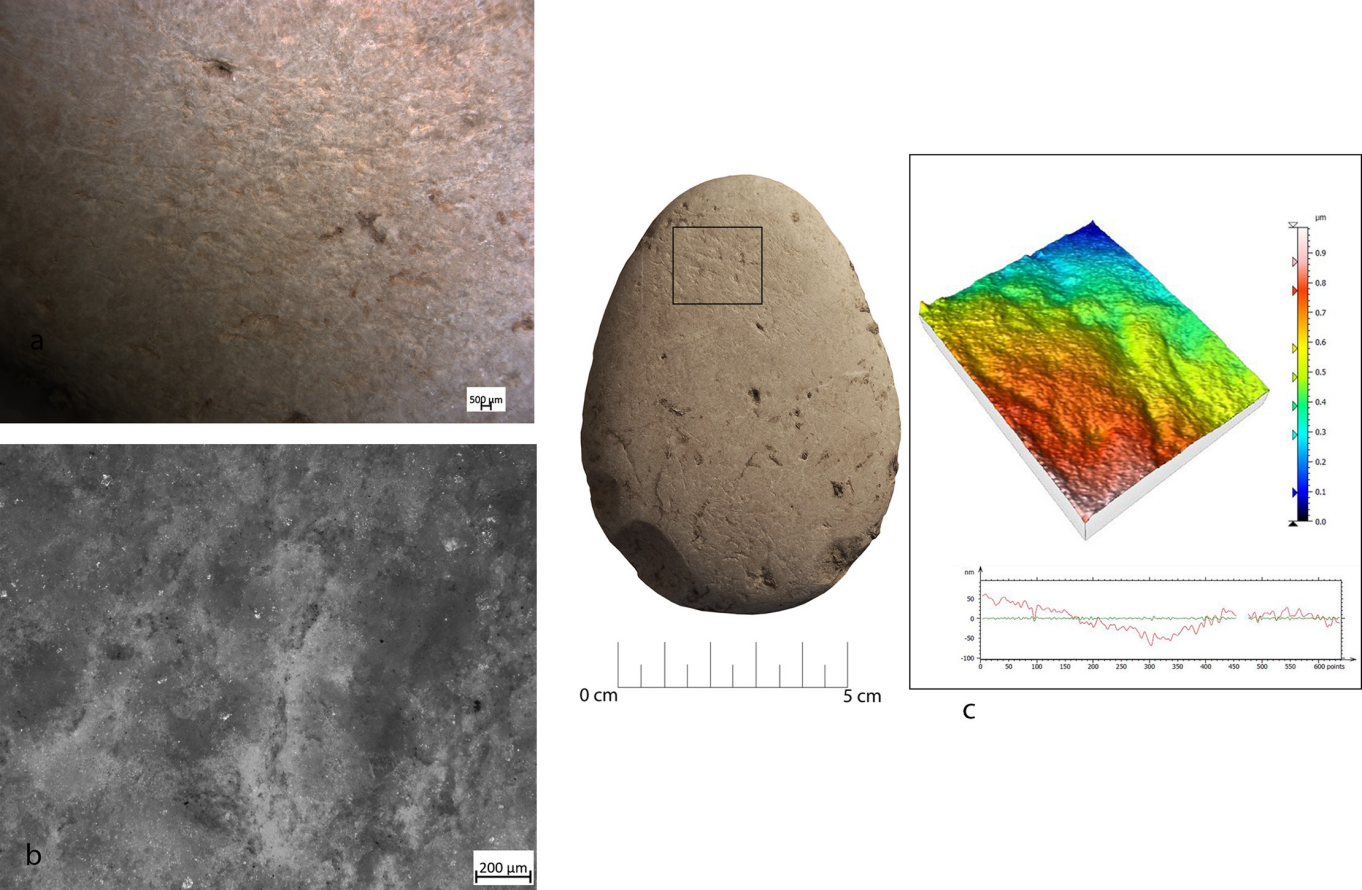
Use wear identified on artefact RF67. (a) Macro-traces (10x), contiguous pits of linear form (half-moon), with rough bottom and triangular section, located on the centre of apical area; (b) micro-traces (200x) are absent; (c) 3D microtopography of the used surface and profile.

**Fig 24:**
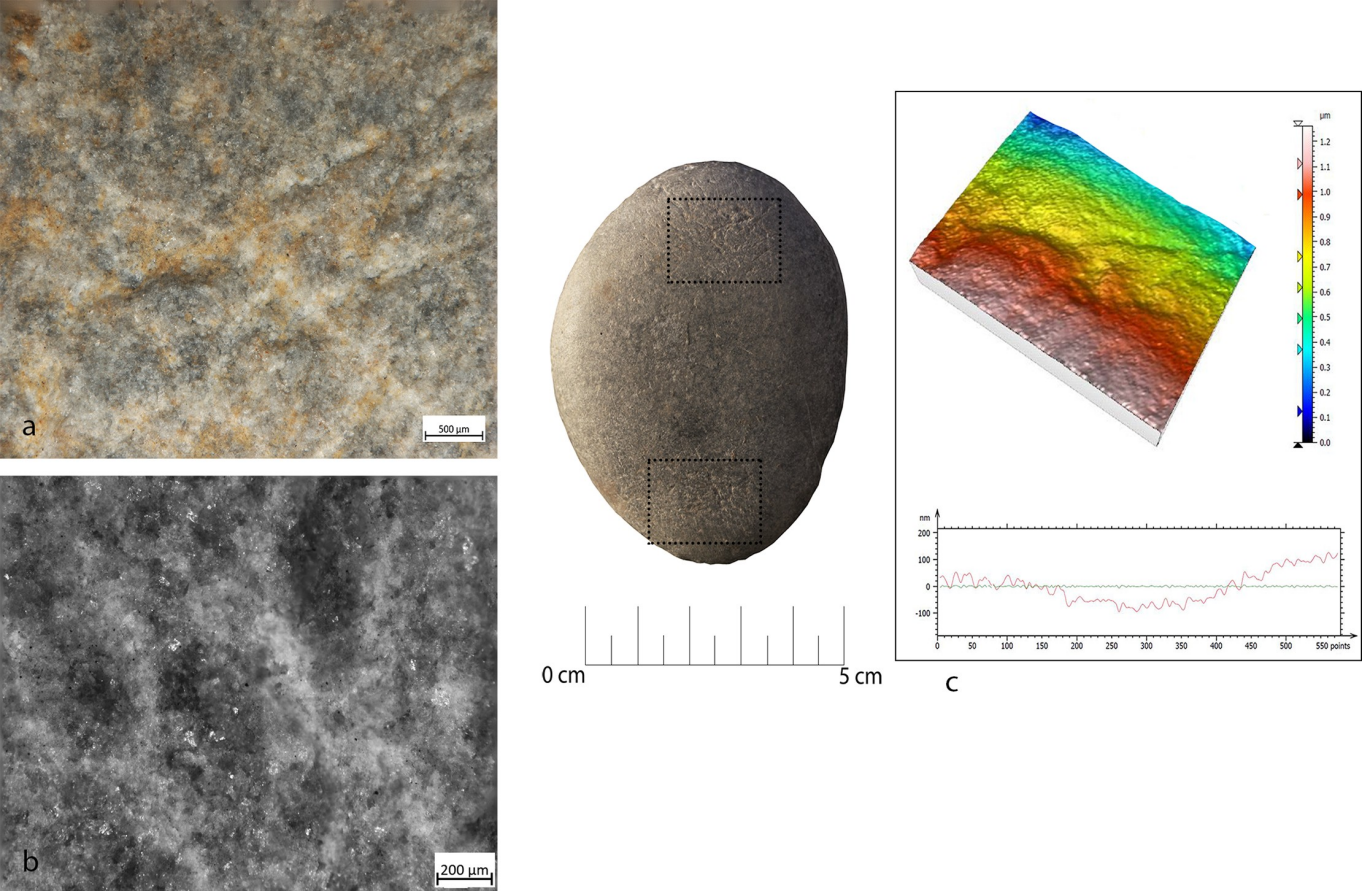
Use wear identified on artefact RF127. (a) Macro-traces (30x), contiguous pits of linear form (half-moon), with rough bottom, located on the centre of apical area; (b) pits with rough bottom (200x); (c) 3D microtopography of the used surface.

Macro-traces observed at the stereo-microscope were represented by pits and striations. However, the pits displayed differences in morphology and location. In two cases (RF67 and RF127) the pits were located on the apices opposite to the flat surfaces (on one or both surfaces). The morphology of the pits was linear (reduced half-moon), with a triangular section. Polishing was not present (**Figs 23 and 24).**

**Fig 25:**
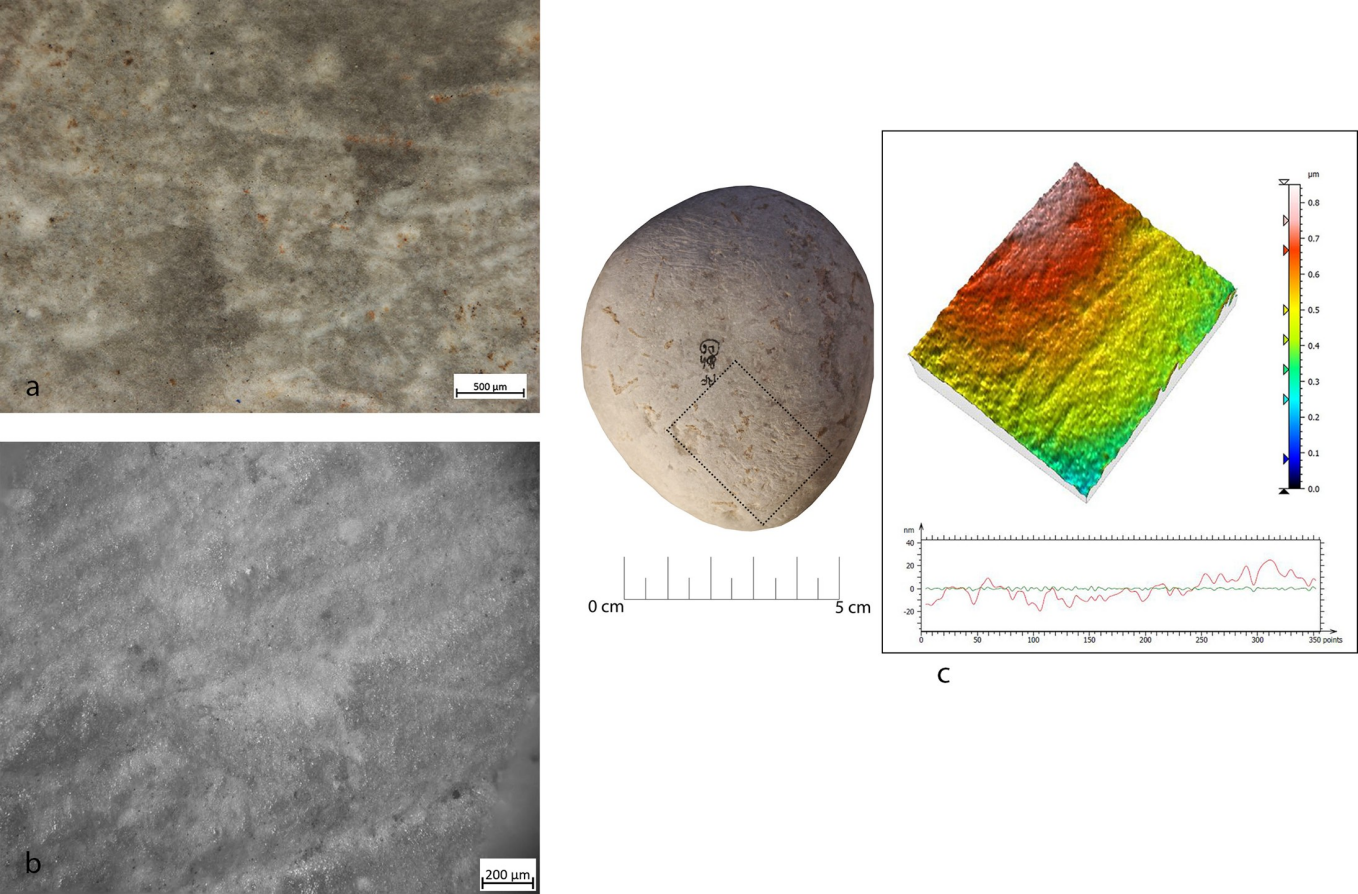
Use wear identified on artefact RF80. (a) Macro-traces (20x), small circular pits associated with long parallel striations, located in apical top with oblique orientation; (b) micro-traces (200x), polishing is absent, a general rounding of the artefact can be observed; (c) 3D microtopography of the used surface.

In two other cases (RF80 and RF92) **(Fig 25**) pits were always located on the flat surfaces of the tool over the apices and were characterized by circular morphology, associated with the presence of long, parallel, superficial and overlapped striations. These traces were very similar to ones observed in the experimental replica used for marginal retouching.

#### GIS analysis—Archaeological sample

As in the case of the experimental replicas, the raw material characterising the archaeological specimens presented in this work was of a homogeneous nature overall.

Specimen RF67 was interpreted through use wear analysis as likely to be a retoucher used to produce scaled retouching based on the presence of traces of use over its apical area, where depressions characterised by a mean slope value of 9.8° were present. The topography of the surface was homogeneous overall with a low to medium degree of surface roughness (TRI mean value 0.00138) along with a low degree of topographic variation as indicated by the VRM mean value (0.0032). Wear generated by use featured a mean perimeter of 9 mm and mean area of 1 mm^2^. As observed on the experimental replica, use related traces were located towards the artefact edge (mean distance 12mm), while their average distance from the tools centre was 20mm. Wear results were well dispersed over the apical area of the retoucher as indicated by the standard deviational ellipse elongation value (1.6 ad).

**Fig 26:**
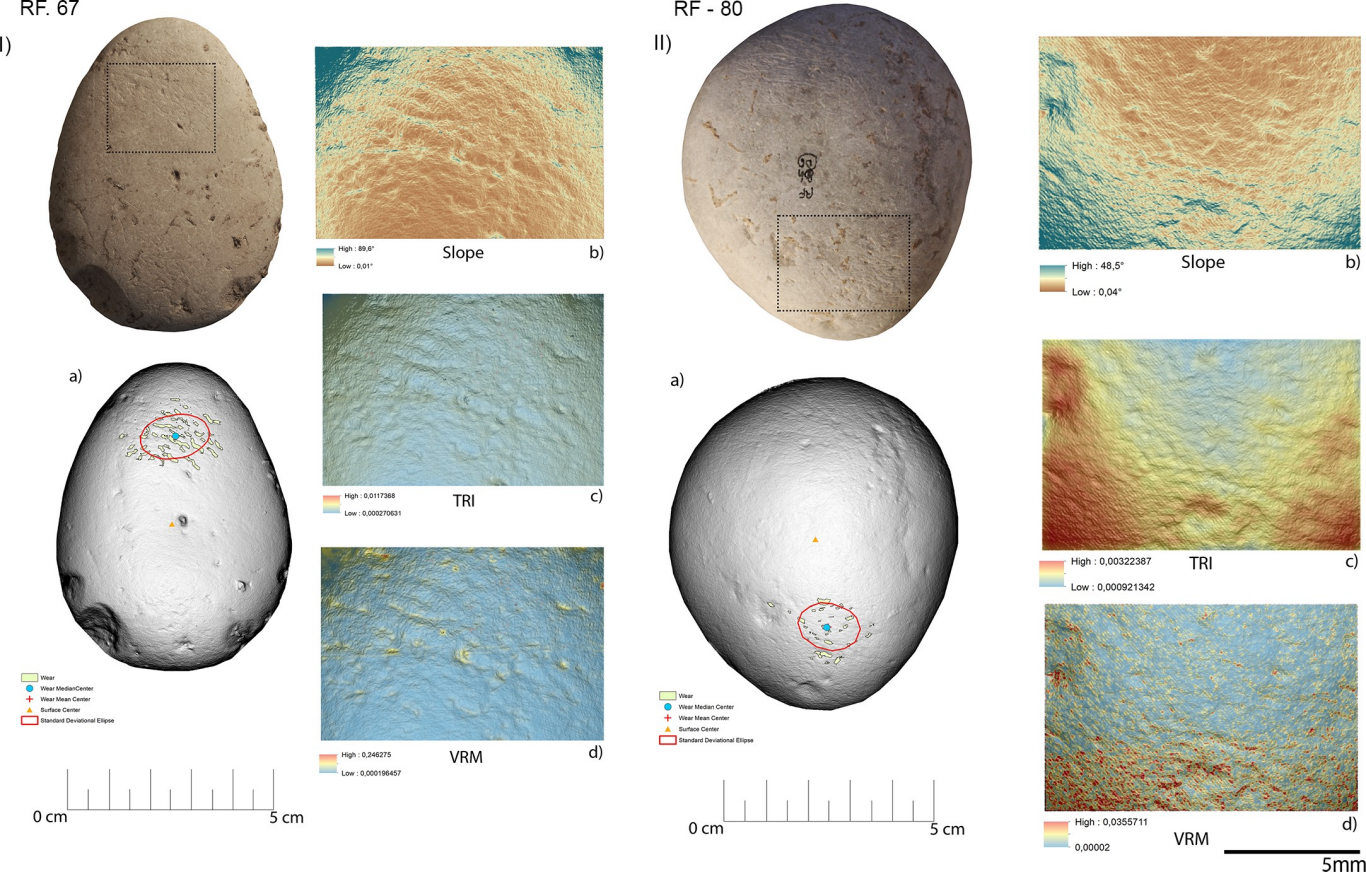
Archaeological items, RF67 (I) and RF80 (II), utilized in scaled retouching and marginal retouching. (a) Spatial distribution of the identified wear; (b) slope; (c) terrain roughness index; (d) vector roughness measure.

Use wear identified on artefact RF80 (**Fig 26II, Table 5**) allowed us to interpret its function as a retoucher utilised for marginal retouching. As in the case of artefact RF67 (**Fig 26I, Table 5**) wear was located over the apical area of the object, where depressions bearing a mean slope value of (13.8°) were visible. The utilised area was characterised by a rough surface (TRI mean value 0.0021) becoming smoother towards the centre of the tool. The same pattern was evinced from VRM (mean value 0.0016), with a higher degree of topographic variation towards the outer portion of the tool apical area and lower values characterising its inner portion. This phenomenon can be explained by the fact that the outer area of the tool’s apex suffered a higher degree of surface crushing compared to its inner area. The traces observed on RF-80 were relatively small with an average perimeter of 2.8 mm and an average area of 0.33 mm^2^. Use related damage was localised nearer the edge of the retoucher (average distance 11 mm) than its centre (mean distance 14 mm). Use wear appeared dispersed over the apical area of the tool as indicated by the standard deviational ellipse value (1.3 ad).

**Table 5:** Morphometric features of the wear identified on the utilised areas of the archaeological specimens.

	RF-67	RF-80	RF-92 (a)	RF-92 (b)	RF-73	RF-127 (a)	RF-127 (b)	RF-127 (c)
**Perimeter (mm)**								
*Minimum*	3.7	1.1	3.7	4	2.8	3.5	3.4	0.1
*Maximum*	18	10	32	14	26	13	10.5	17
*Average*	9	2.8	7	7	7	6.3	6.7	2.7
**Area (mm2)**								
*Minimum*	1	0.1	1	1	1	1	1	1
*Maximum*	4	1.5	10	2	6	2	2	4
*Average*	1	0.3	1	1	1	1	1	1
**Distance from Centre (mm)**								
*Minimum*	13	9.2	2.4	26	0.4	9.5	12	
*Maximum*	29	19	19.4	40	18	20	22	
*Average*	20	14	10	32	9.5	16	17	
**Distance from Edge (mm)**								
*Minimum*	6	6	10	6	13	4.6	4.6	
*Maximum*	18	15	26	19	33	16	14	
*Average*	12	14	20	12	25	10	9	
**Standard Deviational Ellipse**								
*Perimeter (mm)*	42	27	69	50	65	32.7	29	42
*Area (mm2)*	130	55	372	170	330	81	64	180
*Elongation (ad)*	1.6	1.3	0.7	2	1.3	1.3	1.3	2.5
**Used Area (%)**	2	<1	1	1	1	<1	<1	4
**Pits Density (mm2)**	0.01	0.01	0.01	0.01	0.01	0.01	0.01	0.01

**Fig 27:**
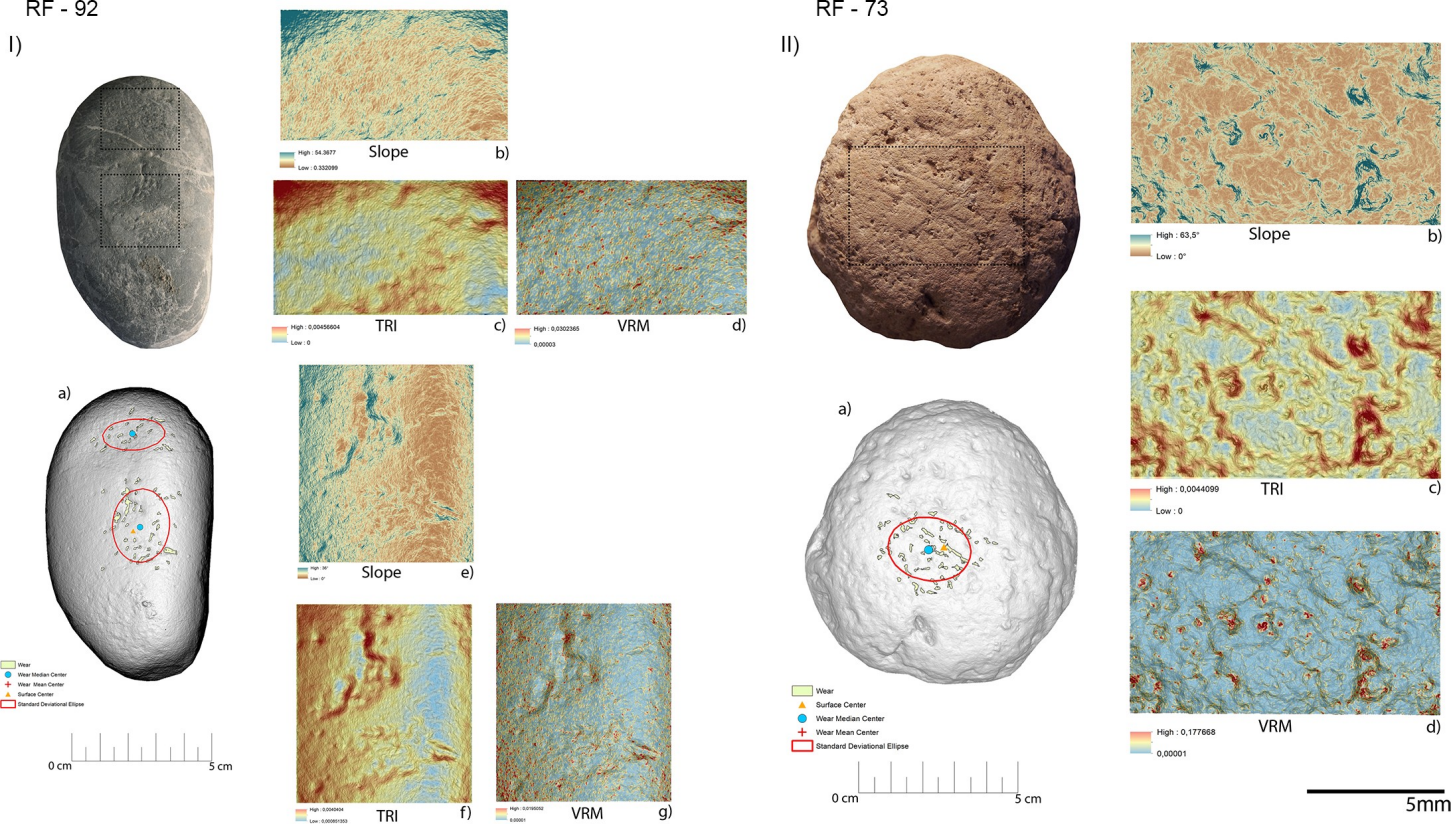
Archaeological items RF92 (I) and RF73 (II) utilised in retouch and percussion activities (RF92) and core ridge adjustment (RF73). (a) Spatial distribution of the identified wear; (b) slope; (c) terrain roughness index; (d) vector roughness measure.

Two distinctive functional areas were identified on artefact RF92 (**Fig 27I, Table 5**), one localised at the centre of the object and one corresponding to its apical area. The wear identified on each of the FAs was related to two different activities, passive percussion (RF-92a) and marginal retouching (RF92b). RF-92a was characterised by the presence of depressions with a mean slope value of 22.6°, while more gentle slopes (mean value 15.6°) characterised the depressions identified over RF92b. The surfaces of both the functional areas exhibited a medium to high degree of roughness, with RF92a exhibiting a TRI mean value of 0.0022 and RF-92b featuring a TRI mean value of 0.0029. A difference between the two surfaces was found in their topographic variability. While RF92a was characterised by a low VRM mean value (0.0008), with the higher values corresponding to the bottom of the traces generated by use, a higher variability characterised RF-92b (VRM mean value 0.0023) where higher values were spread over the entire used surface. Traces observed on the central area exhibited a mean perimeter of 7mm and an average area of 1mm^2^. The damage was located close to the centre of the tool (mean distance 10 mm) and were concentrated, as indicated by the standard deviational ellipse elongation value of 0.7ad.

Use related damage identified on RF92b featured a mean perimeter of 7mm and an average area of 1 mm^2^. Traces were localised in proximity of the tool’s edge (average distance 12mm) and were dispersed over the utilised area (stde elongation 2 ad).

Use wear associated with the adjustment of the core ridges was identified over artefact RF73 (**Fig 27II, Table 5**). The utilised area of the tool was characterised by depressions bearing a mean slope value of 9.6°. Overall the surface topography was characterised by a medium to high degree of roughness (TRI mean value 0.0021) along with a low to medium degree of topographic variability (VRM mean value 0.0014). Traces related to use featured an average perimeter of 7 mm and an average area of 1 mm^2^. Damage was localised near the artefact’s centre (mean distance 10mm) and was moderately dispersed over the used surface (stde elongation 1.3ad).

**Fig 28:**
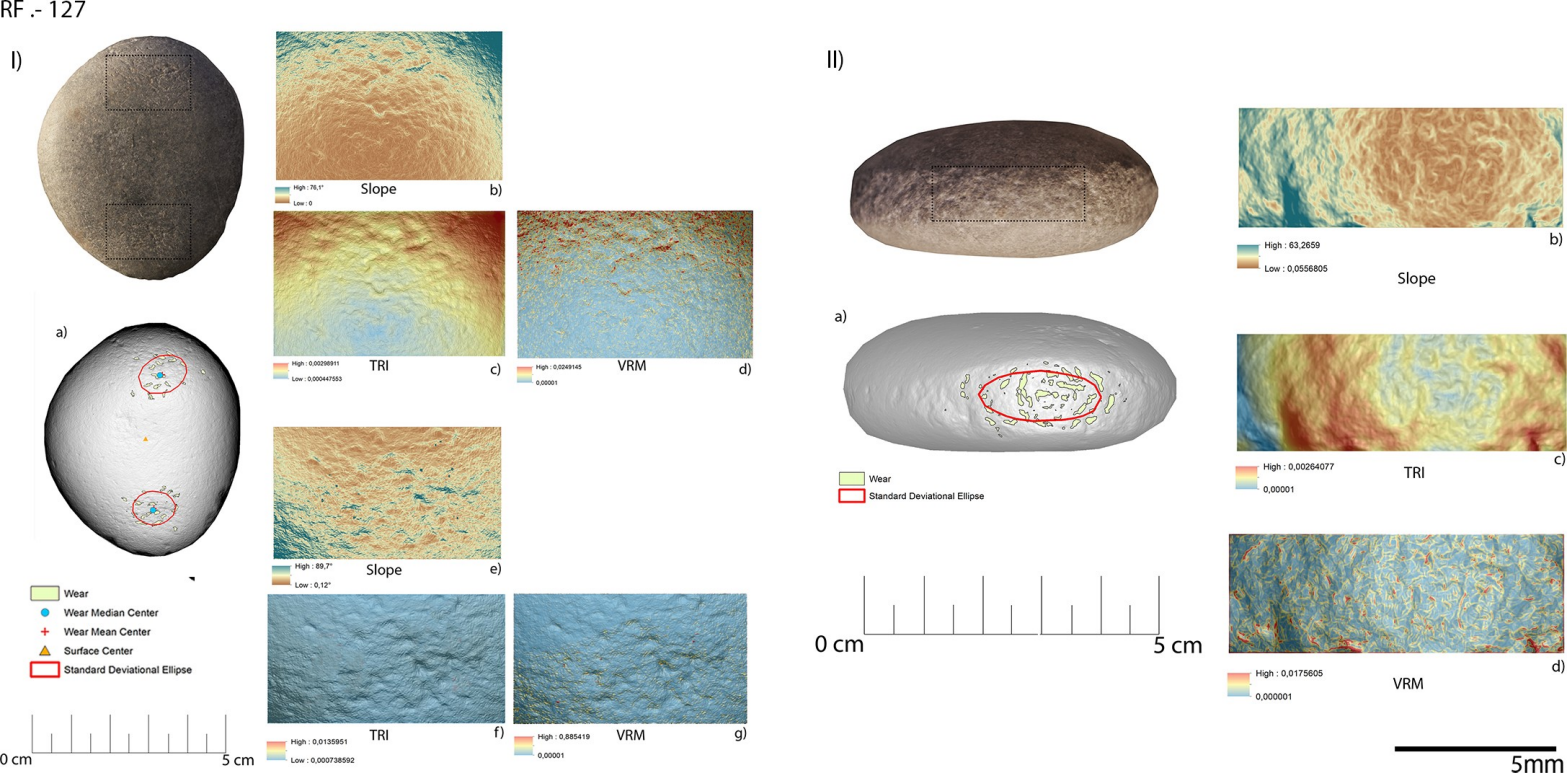
Archaeological items RF127. The surface of the tool (I) has been used in retouch activities while its edge (II) was used to produce bladelets/core adjustment. (a) Spatial distribution of the identified wear; (b) slope; (c) terrain roughness index; (d) vector roughness measure.

**Fig 29:**
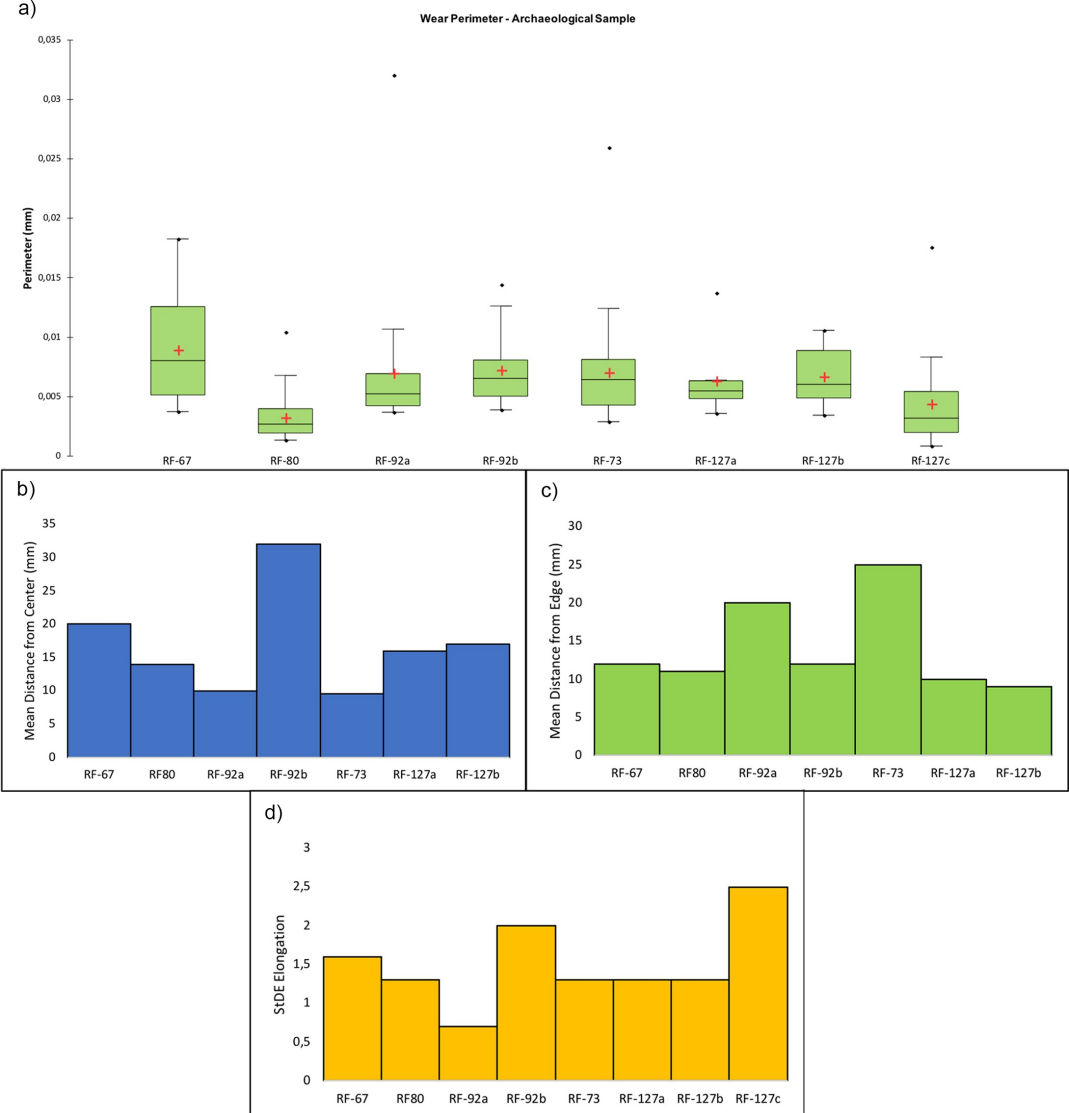
Archaeological specimens. (a) Dimension of the wear identified over the utilized areas; (b) mean distance of the identified wear from the object centre; (c) mean distance of the identified wear from the object edge; (d) dispersion of the identified wear over the tool surface defined by the elongation of the standard deviational ellipse.

Three functional areas were identified on artefact RF127, corresponding to its apices (RF127a; RF127b) (**Fig 28I, Table 5**) and its short edge (RF-127c) (**Fig 28II, Table 5**). The wear identified on the apical area was associated with the production of scaled retouching, while the traces affecting its short edge were related to percussion activity involving the production of blank and core management. The apical area of the tool was characterised by a medium degree of surface roughness: TRI mean value 0.0015 (apical top) and TRI mean value 0.0021 (apical bottom). Both the apices were characterised by a low degree of topographic variability as indicated by the VRM mean values of 0.0012 (apical top) and 0.0011 (apical bottom). Use related damage affecting the top apical area featured a mean perimeter of 6.3 mm and an average area of 1 mm^2^. Similar dimensions were recorded within the traces located on the bottom apical area of the tool (average perimeter 6.7 mm and mean area 1 mm^2^). On both functional areas use related damage was dispersed over the surface as indicated by the recorded standard deviational ellipse value of 1.3 ad. The short edge of RF127 was instead characterised by slightly steeper depressions (mean value 20°) compared to the ones observed over its flat surface. The topography of the surface was moderately rough (TRI mean value of 0.0021) with the lower values coinciding with the area of the edge mostly affected by use related damage. The surface topographic variability was low, given the VRM mean value of 0.0006. The traces identified on the short edge of RF127 exhibited a mean perimeter of 2.7 mm and an average area of 1 mm^2^. They appeared highly dispersed over the utilised surface, as indicated by the high standard deviational ellipse elongation value of 2.5 ad (**Fig 29, Table 5**).

## Discussion

Through the application of a dedicated experimental framework we were able to test the usage, and suitability for the task, of different areas of the hammerstone or retoucher. Use wear analysis, performed at low and high magnification, permitted the definition of the morphological characteristics of wear associated with each of the performed activities. In the study of archaeological samples from Fumane Cave, macro-trace analysis, performed at low magnification, resulted to be more indicative than the observation of micro wear at high magnification, due to the fact that in some cases chemical alteration had prevented the preservation of the micro traces. GIS analysis allowed the investigation of the macro-traces from a quantitative point of view, analysing aspects such as dimensions and spatial distribution of the wear generated by each activity. Moreover, it permitted the collection of data concerning the topographic characteristics (e.g. slope, roughness and topographic variability) of the utilised area of the tool.

Overall, the dedicated experimental framework allowed the isolation of both qualitative and quantitative features concerning use wear deriving from both percussion and retouching activities. The microscopic analysis of the surfaces provided qualitative aspects such as development of polish, micro-striations etc. GIS analysis revealed quantitative data (distance from centre, distance from edge and wear dispersion, this latter defined by the standard deviational ellipse elongation value) concerning the morphometry of use related damage associated to retouching activity, bipolar percussion and core maintenance activities.

**Fig 30:**
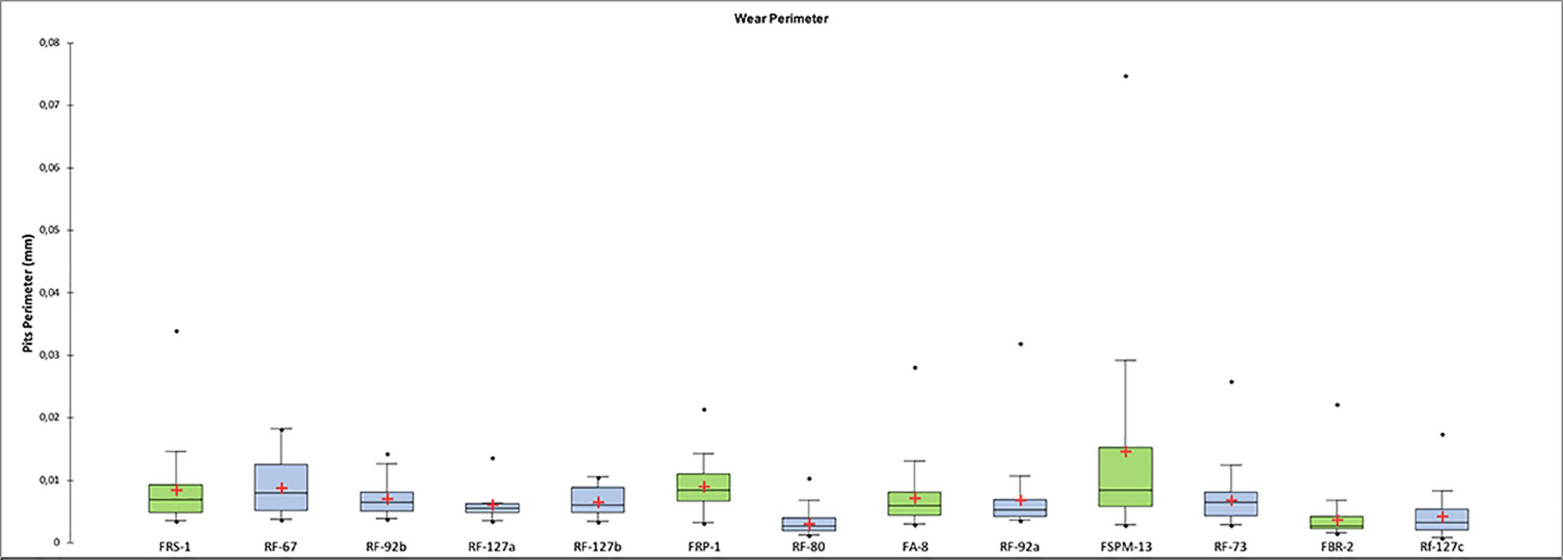
Comparison between the perimeters of the wear observed on the experimental = green, and archaeological = blue specimens.

**Fig 31:**
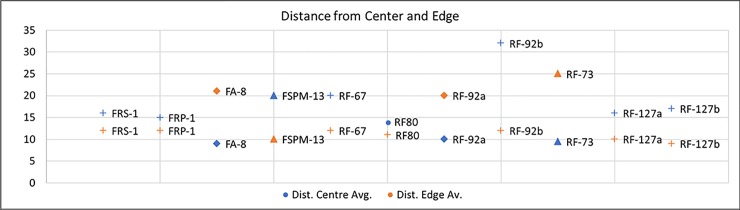
Comparison between the mean distances from the centre and the edge of the tool observed on the experimental and archaeological specimens.

**Fig 32:**
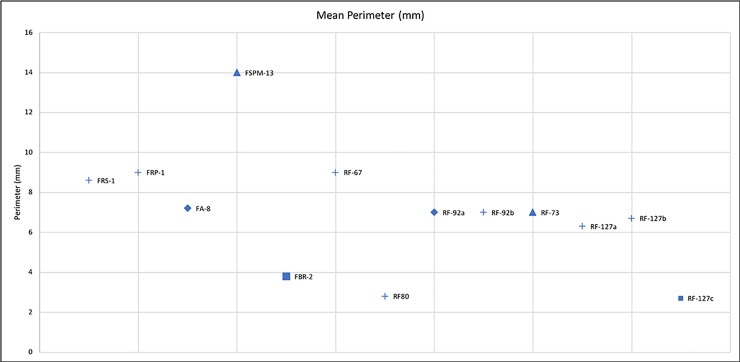
Comparison between the mean perimeter of the wear identified on the experimental and archaeological specimens. In detail, crosses = retouch activities; diamonds = bipolar percussion; triangles = striking platform management; square = bladelets production).

Comparing the experimental and archaeological datasets provided positive results (**Figs 30, 31 and 32**), supporting interpretation derived from use wear analysis. However, on this matter, a note of caution needs to be made. When the dimensions of damage were compared, those of the wear on the experimental replicas resulted to be much larger than those observed on the archaeological materials. This is due to the post depositional alteration affecting the archaeological specimens and leading to an overall rounding and modification of the wear morphology, suggesting that dimensions alone cannot be considered as a diagnostic feature in the interpretation of tool use.

**Fig 33:**
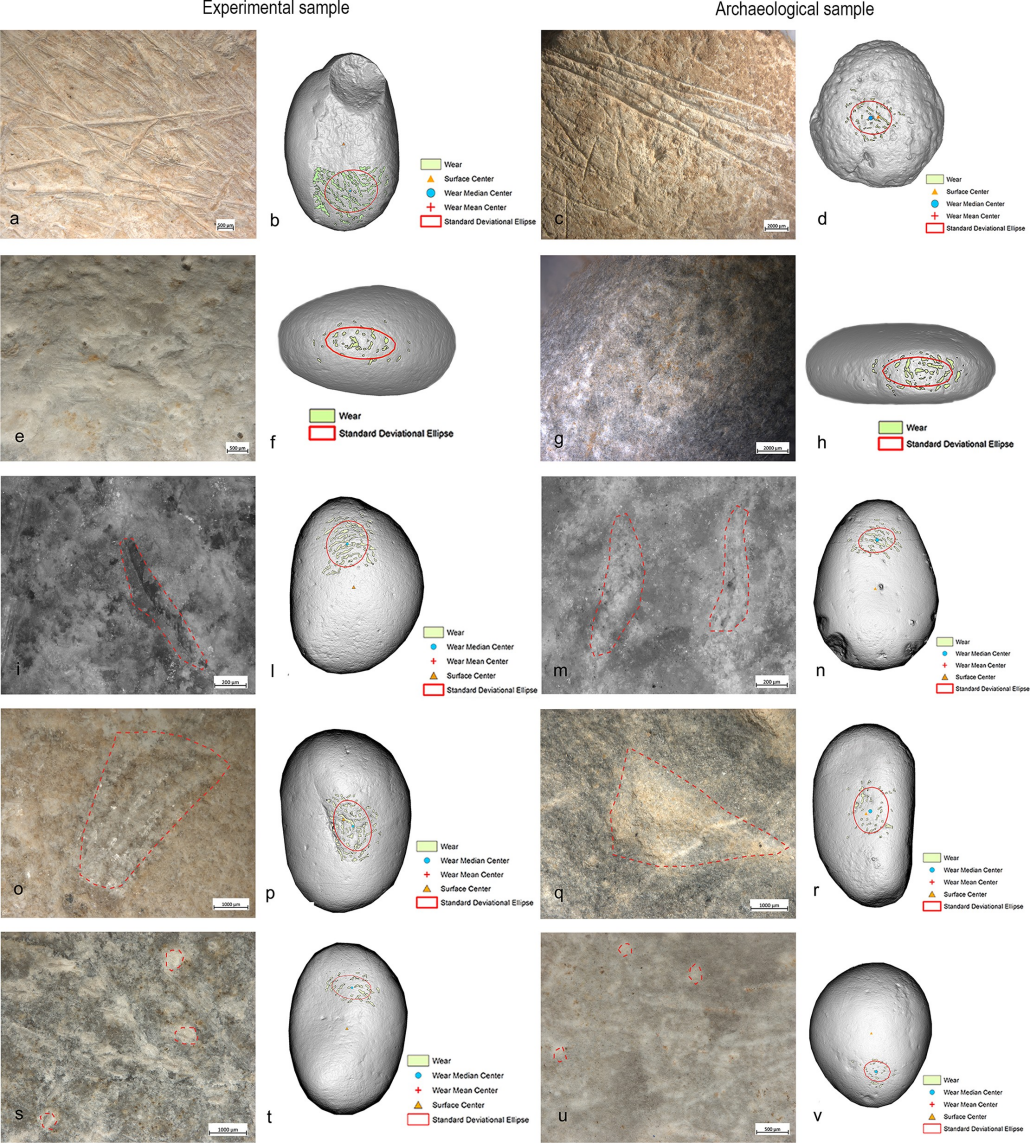
Comparison between the experimental and archaeological use wear and their distribution. (a) Experimental striations (10x) related to the overhang abrasion, localised (b) on the flat surface of the pebble; (c) archaeological striations (10x), localised (d) on the flat surface of the sample; (e) experimental pits (15x) related to core maintenance/bladelets removal, with sub-oval morphology, localised (f) around the short edge of the pebble; (g) archaeological pits (10x) with sub-oval morphology, (h) localised around the short edge of the sample; (i) experimental pits (40x) related to scaled retouching, with linear (half-moon) morphology, rough bottom, localised (l) on the apices of the flat surface; (m) archaeological pits (40x) with linear (half-moon) morphology, rough bottom, localised (n) on the apices of the flat surface; (o) experimental pits (20x), related to the anvil used for the flakes detachment, with sub-triangular morphology, localised (p) in the centre of the pebble; (q) archaeological pits (20x), with sub-triangular morphology, localised (r) in the centre of sample; (s) experimental circular pits associated with the striations (20x), related to marginal retouching, localised (t) on the apices of the flat surface with oblique orientation; (u) archaeological circular pits and striations associated (20x), localised on the apices of the flat surface with oblique orientation.

Within the Fumane Cave macro-lithic sample, several implements exhibited use patterns resembling the ones recorded on the experimental replicas used in scaled and marginal retouching. In particular, artefacts RF67 and RF127 have been interpreted as retouchers used for scaled retouching, while the apical area of artefact RF92 and RF80 exhibited use wear features which led to their interpretation as retouchers used to produce marginal retouching. On 4 archaeological artefacts coming from Fumane Cave, the presence of overlapping pits over the short edges of the tools (RF127, RF138) and of deep long striations affecting the flat surface of the implements (RF73, RF37) led to the interpretation of the artefacts as hammerstones used in both blank production and core maintenance activities. Wear patterns similar to the ones associated with bipolar percussion have been identified on the central surface area of artefact RF92 leading to the interpretation of the use of its central area as an anvil (**Fig 33**). Our results enabled the identification of specific functional patterns related to the use of hammerstones and retouchers at Fumane Cave. We have been able to isolate specific patterns both regarding the morphology of the wear, its spatial distribution and the topography of the used area associated with each of the activities performed. This permitted the placing of the Protoaurignacian and Aurignacian macro-tools of Fumane Cave into specific stages of the production process of chipped tools. Our analysis underlined the high efficiency of the Fumane cave macro-tools in activities concerning core maintenance, blank production and tool retouching. The use of these implements in advanced stages of core maintenance and blank production is suggested by the absence of artefacts bearing traces associated with the initial stage of core reduction. Furthermore, the analysis of the retouchers suggests relevant behavioural insights regarding the choice of objects with specific features (i.e. different types of limestones, soft or compact; the morphological features that favours the success of the product;). Moreover, the analysis of wear from a morphological and spatial point of view permitted to formulate a preliminary hypothesis, that will be confirmed in the future, under which the archaeological tools were used employing two preferential gestures, perpendicular and oblique, involved in the production of scaled, marginal and abrupt retouches. The experimental results showed how, adopting a scaled retouch, it was possible not only to package or maintain formal tools such as end-scrapers, but also to delineate the lateral edges of some thicker blades. On the contrary, marginal and abrupt retouch was mainly used to transform the flake/blade edges. Retouching on an anvil, and edge abrasion techniques aimed at bladelet retouching, currently do not match with the archaeological traces and, following our results, it was difficult to use the flat surface of the retoucher to perform the former activity. The absence of these types of use wear does not exclude that other raw materials and techniques have been used in the production of the Dufour and pointed bladelets at Fumane Cave.

## Conclusion

Given the lack of functional studies focused on the uses of hammerstones and retouchers, the combined approach presented here enhances our current knowledge of this specific kind of tool. This approach provides data not only related to the use of the tools at the site (e.g. [128]) but also involving the gestures and ergonomic choices characterising the Protoaurignacian and Aurignacian human groups of Fumane Cave. As emphasized by Bracco et al. [129] the reconstruction of gesture plays a major role within the analysis of technical processes. The traces of prehension observed and documented during the experimental phase, and evidenced in the archaeological sample, reveal that in Fumane Cave there were different ways of handling the objects. However, it is evident that the study of the variables on the modalities of prehension requires the formulation of a specific experimental protocol.

The preliminary study conducted here showed the potentials of an integrated method applied to the study of prehistoric macro-lithic tools, which can be successfully increased in the future with the support of a broader experimental collection. Our results emphasize the importance of the combination of qualitative (use wear) and quantitative (GIS analysis) approaches which can be applied to a variety of tool categories, providing new data enhancing not only our knowledge regarding the use of ancient Palaeolithic or Mesolithic tools but also, in a broader way, our understanding of ancient human behaviour.
